# Supplementary material for: The applicability of basic preventive measures of the pandemic COVID-19 and associated factors among residents in Guraghe Zone
Source: PLoS One. 2021 Aug 25;16(8):e0256598. doi: 10.1371/journal.pone.0256598 (PMC8386865; doi:10.1371/journal.pone.0256598)
Supplement: Attachment — Submitted filename: PONE-D-21-05518_R2 with comments.pdf [file pone.0256598.s005.pdf]

PLOS ONE

The applicability of basic preventive measures of the pandemic COVID-19 and associated factors among residents in Guraghe Zone

--Manuscript Draft--

**Manuscript Number:**	PONE-D-21-05518R2
**Article Type:**	Research Article
**Full Title:**	The applicability of basic preventive measures of the pandemic COVID-19 and associated factors among residents in Guraghe Zone
**Short Title:**	Preventive measures of the pandemic COVID-19 and associated factors
**Corresponding Author:**	Samuel Dessu, Masters<br>Wolkite University<br>Wolkite, SNNPR ETHIOPIA
**Keywords:**	COVID-19; preventive measures; applicability; Guraghe Zone; pandemic

Abstract:

Introduction

Internationally, countries have reacted to the COVID-19 outbreak by introducing key public health non-pharmaceutical interventions to protect vulnerable population groups. In response to COVID-19, the Government of Ethiopia has been taking a series of policy actions beyond public health initiatives alone. The novelty of this disease, along with its uncertainties, makes it critical for health authorities to plan appropriate strategies to prepare and manage the public. Therefore, this study was aimed to determine the applicability of basic preventive measures of the pandemic COVID-19 and associated factors among the residents of Guraghe Zone from 18 th to 29 th September, 2020.

Methods

Community based cross sectional study was conducted at Guraghe zone from 18 th to 29 th September, 2020. Systematic random sampling methods were applied among the predetermined 634 samples. Data were entered into Epi-data version 3.01 and exported to SPSS version 25 for analysis. Both bivariate and multivariable logistic regression model were fitted to assess the association between outcome and explanatory variables. Variables which had p-value less than 0.25 in bivariate analysis were considered candidates for multivariable logistic regression model. P-value ≤ 0.05<0.05 was used as a cutoff point to determine statistical significance in multiple logistic regressions for the final model.

Result

In this study, 21.9%(95%CI: 18.9~~0~~, 25.1~~0~~) of the respondents have good knowledge, 94.2%(95%CI: 92.3~~0~~, 95.9~~0~~) had a favorable attitude and 17.7~~0~~% (95% CI: 14.7~~0~~, 20.5~~0~~) apply basic preventive measures towards the prevention of the pandemic COVID-19. In addition, being rural resident (AOR: 4.78~~,~~; 95%CI: 2.5, 8.9), being studied grade 1-8 (AOR: 3.7; 95%CI: 1.7, 7.9), being a farmer (AOR: 4.1; 95%CI: 1.25, 13.35), currently not married (AOR: 2.2, 95%CI: 1.24, 4.06), having family size 1-3(AOR: 6.5; 95%CI: 3.21, 3.35), have no diagnosed medical illness (AOR: 6.4; 95%CI: 3.85, 10.83) and having poor knowledge (AOR: 3.5; 95%CI: 1.60, 7.40) were factors which are statistically significant in multivariable logistic regression model.

Conclusion

The applicability of the pandemic COVID -19 preventive measures was too low, which indicate that the Zone is at risk for the infection and, being rural residence, having lower educational level, being a farmer, being currently not married, having lower family size, not having diagnosed medical illnesses and poor knowledge level were factors associated with the applicability of the preventive measures of the pandemic COVID-19. In addition, awareness creation should be in practice at all levels of the community especially lower educational classes and rural residents.

**Order of Authors:**	Samuel Dessu, Masters
	Tadesse Tsehay
	Tadele Girum
	Abebe Timerga
	Mamo Solomon
	Baye Tsegaye
	Mulugeta Geremew
	Biru Migora
	Yibeltal Mesfin
	Fisha Alebel
	Omega Tolosa
	Shegaw Tesfa
	Abdurezak Kemal
	Fedila Yasin
**Response to Reviewers:**	Both concdrns were addressed and corrected.
**Additional Information:**	
**Question**	**Response**
**Financial Disclosure**<br><br>Enter a financial disclosure statement that describes the sources of funding for the work included in this submission. Review the submission guidelines for detailed requirements. View published research articles from *PLOS ONE* for specific examples.<br><br>This statement is required for submission and **will appear in the published article** if the submission is accepted. Please make sure it is accurate.	The authors received no specific funding for this work.

**Unfunded studies**<br>Enter: *The author(s) received no specific funding for this work.*<br><br>**Funded studies**<br>Enter a statement with the following details:<br>• Initials of the authors who received each award<br>• Grant numbers awarded to each author<br>• The full name of each funder<br>• URL of each funder website<br>• Did the sponsors or funders play any role in the study design, data collection and analysis, decision to publish, or preparation of the manuscript?<br>• **NO** - Include this sentence at the end of your statement: *The funders had no role in study design, data collection and analysis, decision to publish, or preparation of the manuscript.*<br>• **YES** - Specify the role(s) played.<br><br>* typeset	
**Competing Interests**<br><br>Use the instructions below to enter a competing interest statement for this submission. On behalf of all authors, disclose any competing interests that could be perceived to bias this work—acknowledging all financial support and any other relevant financial or non-financial competing interests.<br><br>This statement is required for submission and **will appear in the published article** if the submission is accepted. Please make sure it is accurate and that any funding sources listed in your Funding Information later in the submission form are also declared in your Financial Disclosure statement.<br><br>View published research articles from *PLOS ONE* for specific examples.	The authors have declared that no competing interests exist.

**NO authors have competing interests**<br><br>Enter: *The authors have declared that no competing interests exist.*<br><br>**Authors with competing interests**<br><br>Enter competing interest details beginning with this statement:<br><br>*I have read the journal's policy and the authors of this manuscript have the following competing interests: [insert competing interests here]*<br><br>* typeset	
**Ethics Statement**<br><br>Enter an ethics statement for this submission. This statement is required if the study involved:<br><br>• Human participants<br>• Human specimens or tissue<br>• Vertebrate animals or cephalopods<br>• Vertebrate embryos or tissues<br>• Field research<br><br>Write "N/A" if the submission does not require an ethics statement.<br><br>General guidance is provided below. Consult the submission guidelines for detailed instructions. **Make sure that all information entered here is included in the Methods section of the manuscript.**	Ethical clearance was obtained from Wolkite University College of medicine and health sciences ethical review board and permission letter was obtained from the Guraghe Zone health department and the corresponding Woreda health care administrative offices. A written consent was obtained directly from the respondents. All the necessary measures were taken to maintain and assure the confidentiality and all benefits of the patients.

Powered by Editorial Manager® and ProduXion Manager® from Aries Systems Corporation

**Format for specific study types**<br><br>**Human Subject Research (involving human participants and/or tissue)**<br>• Give the name of the institutional review board or ethics committee that approved the study<br>• Include the approval number and/or a statement indicating approval of this research<br>• Indicate the form of consent obtained (written/oral) or the reason that consent was not obtained (e.g. the data were analyzed anonymously)<br><br>**Animal Research (involving vertebrate animals, embryos or tissues)**<br>• Provide the name of the Institutional Animal Care and Use Committee (IACUC) or other relevant ethics board that reviewed the study protocol, and indicate whether they approved this research or granted a formal waiver of ethical approval<br>• Include an approval number if one was obtained<br>• If the study involved *non-human primates*, add *additional details* about animal welfare and steps taken to ameliorate suffering<br>• If anesthesia, euthanasia, or any kind of animal sacrifice is part of the study, include briefly which substances and/or methods were applied<br><br>**Field Research**<br><br>Include the following details if this study involves the collection of plant, animal, or other materials from a natural setting:<br>• Field permit number<br>• Name of the institution or relevant body that granted permission	
**Data Availability**<br><br>Authors are required to make all data underlying the findings described fully available, without restriction, and from the time of publication. PLOS allows rare exceptions to address legal and ethical concerns. See the PLOS Data Policy and FAQ for detailed information.	Yes - all data are fully available without restriction

A Data Availability Statement describing where the data can be found is required at submission. Your answers to this question constitute the Data Availability Statement and **will be published in the article**, if accepted.<br><br>**Important:** Stating 'data available on request from the author' is not sufficient. If your data are only available upon request, select 'No' for the first question and explain your exceptional situation in the text box.<br><br>Do the authors confirm that all data underlying the findings described in their manuscript are fully available without restriction?	
**Describe where the data may be found in full sentences. If you are copying our sample text, replace any instances of XXX with the appropriate details.**<br><br>• If the data are **held or will be held in a public repository**, include URLs, accession numbers or DOIs. If this information will only be available after acceptance, indicate this by ticking the box below. For example: *All XXX files are available from the XXX database (accession number(s) XXX, XXX.)*.<br>• If the data are all contained **within the manuscript and/or Supporting Information files**, enter the following: *All relevant data are within the manuscript and its Supporting Information files.*<br>• If neither of these applies but you are able to provide **details of access elsewhere**, with or without limitations, please do so. For example:<br><br>*Data cannot be shared publicly because of [XXX]. Data are available from the XXX Institutional Data Access / Ethics Committee (contact via XXX) for researchers who meet the criteria for access to confidential data.*<br><br>*The data underlying the results presented in the study are available from (include the name of the third party*	All relevant data are within the manuscript and its Supporting Information files.

and contact information or URL).<br>• This text is appropriate if the data are owned by a third party and authors do not have permission to share the data.<br><br>* typeset	
Additional data availability information:	

Manuscript
Click here to access/download;Manuscript;Revised Manuscript.docx

The applicability of basic preventive measures of the pandemic COVID-19 and associated factors among residents in Guraghe Zone

Samuel Dessu[1]*, Tadesse Tsehay[2], Tadele Girum[1], Abebe Timerga[3], Mamo Solomon[2], Baye Tsegaye[2], Mulugeta Geremew[4], Biru Migora[4], Yibeltal Mesfin[5], Abdurezak Kemal[1], Fisha Alebel[2], Omega Tolosa[2], Shegaw Tesfa[2], Fedila Yasin[1]

[1]Department of Public Health, College of Medicine and Health Sciences, Wolkite University, Wolkite, Ethiopia
[2]Department of Nursing, College of Medicine and health Sciences, Wolkite University, Wolkite, Ethiopia
[3]Department of Biomedical Sciences, College of Medicine and health Sciences, Wolkite University, Wolkite, Ethiopia
[4]Department of Statistics, College of Natural and Computational Sciences, Wolkite University, Wolkite, Ethiopia
[5]Department of Midwifery, College of Medicine and health Sciences, Wolkite University, Wolkite, Ethiopia

Correspondence to Samuel Dessu [Email: dessusamuel@yahoo.com, Tel: +251910883594] & Tadesse Tsehay [Email: tadesse200912@gmail.com, Tel: +251961818585]

Abstract

Introduction: Internationally, countries have reacted to the COVID-19 outbreak by introducing key public health non-pharmaceutical interventions to protect vulnerable population groups. In response to COVID-19, the Government of Ethiopia has been taking a series of policy actions beyond public health initiatives alone. Therefore, this study was aimed to assess the applicability of basic preventive measures of the pandemic COVID-19 and associated factors among the residents of Guraghe Zone from 18th to 29th September, 2020.

Methods: Community based cross sectional study was conducted at Guraghe Zone from 18th to 29th September, 2020. Systematic random sampling method was applied among the predetermined 634 samples. Variables which had p-value less than 0.25 in bivariate analysis were considered as candidate for multivariable logistic regression model. P-value $\leq$ 0.05 was used as a cutoff point to determine statistical significance in multiple logistic regressions for the final model.

Result: In this study, 17.70% (95% CI: 14.70, 20.50) of the respondents apply the basic preventive measures towards the prevention of the pandemic COVID-19. In addition, being rural resident (AOR: 4.78,; 95%CI: 2.5, 8.9), being studied grade 1-8 (AOR: 3.7; 95%CI: 1.7, 7.9), being a farmer (AOR: 4.1; 95%CI: 1.25, 13.35), currently not married (AOR: 2.2, 95%CI: 1.24, 4.06), having family size 1-3(AOR: 6.5; 95%CI: 3.21, 3.35), have no diagnosed medical illness (AOR: 6.4; 95%CI: 3.85, 10.83) and having poor knowledge (AOR: 3.5; 95%CI: 1.60, 7.40) were factors which are statistically significant in multivariable logistic regression model.

Conclusion: Despite the application of preventive measures and vaccine delivery, the applicability of the pandemic COVID-19 preventive measures was too low, which indicate that the Zone is at risk for the infection. Rural residents, those who have lower educational level, farmers, non-marrieds, those who have lower family size, those who have diagnosed medical illnesses and those who have poor knowledge were prone to the infection with the pandemic COVID-19 due to the lower practice of applying the basic preventive measures. In addition, awareness creation should be in practice at all levels of the community especially lower educational classes and rural residents.

Key words: COVID-19; Preventive measures; Applicability; Guraghe Zone; Pandemic

Introduction

The pandemic of coronavirus disease 2019 (COVID-19) started in December 2019 in Wuhan, China [1]. Currently, the virus causing the disease is approaches each continent throughout the world and continues to spread at an alarming rate [2].

Globally, countries have acted up on the COVID-19 outbreak through introducing key public health non-pharmaceutical interventions to protect vulnerable population groups [3]. Lock down, washing hands frequently using soap in mashing for more than 20 seconds, maintain physical distancing, stay informed and follow advice given by healthcare professionals and seeking medical advice if develop cough or fever or experience difficulty of breathing and call in advance the center assigned for COVID-19 response were the recommended measures to reduce the transmission of COVID-19 by world health Organization (WHO) [4].

COVID-19 can results in a severe national problems. In addition; it results in severe psychological, economic crisis across the globe [5, 6, 7, 8]. As the disease progressed, concerns regarding health, economy, and livelihood increased day-to-day. The findings of the pandemic's impact on these issues could help inform health officials and the public to provide mental health interventions to those who are in need [8].

The first case of COVID-19 in Ethiopia was detected on 13th March 2020 involving two tourists from Japan [1]. In accordance with the applied case definition and testing strategies, till 8th of April, 2021, the number of peoples infected with the pandemic COVID-19 in the world was 132,730,691, of them, 3,148, 358 were in Africa and of them, 221,544 were in Ethiopia. Among them, globally 2,880,726 were died, of them, 114,610 were in Africa and of them, 3,058 were in Ethiopia [4].

In response to COVID-19, the Government of Ethiopia has been taking a series of policy actions beyond public health initiatives alone [9]. These include closing schools, restricting use of public transportation, banning large meetings, and suspending sporting and religious gatherings. A state of emergency has been put in effect and staying at home and working from there has been strongly advised [10]. On March 2021, Ethiopia has received 2.184 million doses of the Astra Zeneca COVID-19 vaccine via the COVAX facility and the country is on immunizing the frontline workers and the most susceptible individuals [11].

Despite the implementation of such strategies, the occurrence of the pandemic COVID-19 is still increasing. Therefore, this study focus on the evaluation of the applicability of basic prevention methods of the pandemic COVID-19 may help to the populations at risk to reduce the risk of mortality.

Methods and materials

Study Design, Area and Period

A community based cross-sectional study was conducted at Guraghe Zone from 18th to 29th September, 2020. Guraghe Zone is one of the 13 Zones encountered at South Nations Nationalities and Peoples regional state (SNNPR). Wolkite town is its administrative sit and located at 158 km south from Addis Ababa. Based on the 2007 Ethiopian national population and housing census, the population of the Zone is projected to be about 1,609,908 and 51.38% are females. Administratively the Zone is divided in to 16 districts (Woredas) and 4 city administrations.

Populations

Populations

In this study, all the residents at the Guraghe Zone were considered as a source population and all the selected populations in the Guraghe Zone from 18th to 29th September, 2020 from the source population were termed as study populations.

Inclusion and exclusion criteria

All the ambulatory residents of the Guraghe Zone were included into the study and residents of Guraghe Zone who cannot able to communicate (unable to listen and talk) and children who have age less than 15 were excluded from the study.

Sample size determination and sampling procedures

The required sample size was determined using formula for single population proportion formula n = $\frac{z_{\frac{\alpha}{2}}{}^{2}P(1-P)}{d^{2}}$, Where n denotes the sample size, $z_{\frac{\alpha}{2}}$ denotes the reliability coefficient of standard

error, at 5% level of significance (which is 1.96), P = proportion of residents applicability of basic preventive measures at Guraghe zone (Which is 50% because there was no study conducted before) and d = margin of error. Therefore, the calculated sample size was 384. Using design effect for the sample in using interclass correlation (δ) = 1.5, it became 576. Through considering 10% for non-response rate, the final sample size for the study was 634.

Among the 20 districts found in Guraghe Zone, 7 districts (Abeshge Woreda, Emdebir town administration, Enemor Woreda, Edja woreda, Gumer Woreda, Meskan woreda and Wolkite town administration) were randomly selected using lottery method. From town administrations, four sub cities (two sub cities from Emdebir town administration and two sub cities from Wolkite town administration) were randomly selected using lottery method. Again from the randomly selected rural districts, 23 kebelles were randomly selected (Three kebelles from Abeshge Woreda, five Kebelles from Enemor Woreda, Four Kebelles from Edja Woreda, Five Kebelles from Gumer woreda and six kebelles from Meskan woreda).

Finally, the calculated sample size was proportionally allocated to the randomly selected districts (Abeshge (28), Emdeber (31), Enemor (156), Edja (121), Gumer (114), Meskan (137) and Wolkite town administration (47)) and the households from each district were selected using systematic random sampling method. Within each of the selected household, one individual was taken, primarily the house hold head (father) or the mother or kids based on the hierarchy (eldest to youngest).

Study variables

The dependent variable for this study was the applicability of basic COVID-19 preventive measures and the independent variables were socio demographic variables (age, sex, residence, educational status, occupation, marital status, religion and estimated monthly income), knowledge status of the respondents, attitude towards COVID-19, availability of Mass media and diagnosed medical health problem (Comorbidity)

Operational definitions

High knowledge: If the respondent answers at least 11 of the 14 knowledge assessment questions correctly [12].

Moderate knowledge: If the respondent answers at least nine of the 14 knowledge assessment questions correctly [12].

Poor knowledge: If the respondent responds $\leq$ 8 knowledge assessment questions correctly [12].

Good knowledge: If the respondent responds $\geq$ 9 knowledge assessment questions correctly [12].

Proper hand washing practice: If the respondent wash his/her hands using soap and water in mashing his hands for more than 20 seconds or using hand sanitizers [13].

Favorable attitude towards COVID-19: a mean attitude score $\geq$30 [12].

Unfavorable attitude towards COVID-19: a mean attitude score less than 30 [12].

Apply basic preventive measures: if the respondent applies complete lockdown (stay at home) or reasonable exit in wearing mask appropriately, proper hand washing practice and keeps physical distance [13].

Data collection tool and procedures

Data collection tool was adapted from WHO resources and similar studies. Initially, it was prepared in English and was translated to Amharic by language experts to ensure consistency. Data were collected using structured interviewer administered questionnaires. Data were collected by 15-experienced BSC nurses and were supervised by three Msc holders in health science through the entire data collection process.

The knowledge questions have 14 items. These items include the participant knowledge about clinical presentations (items 1–4), transmission routes (items 5–8) and prevention and control (items 9–14) of COVID-19. Participants were given “true,” “false,” or “I don’t know” response options to these items. A correct response to an item were assigns 1 point, while an incorrect/don’t know response assigned 0 points. The maximum total score ranged from 0–14, with a higher score indicating better knowledge about COVID-19.

To measure attitudes towards COVID-19, 10 items (with minimum score 10 and maximum score 50) were used. Response of each item was recorded on 5-point Likert scale as follows strongly disagree (1-point), disagree (2-point), neutral (3-point), agree (4-point), and strongly agree (5-point). The overall level of attitude was categorized using original Bloom’s cut-off point, as

positive if the score was 80-100%(40-50), neutral if the score was 60-79% (30-40) and negative if the score was less than 60 (10-29). A mean score $\geq$30 was carried out as a favorable attitude and a score less than 30 indicated an unfavorable attitude toward COVID-19.

Data quality management

Data quality was assured by caring out careful design of data collection tool and appropriate modification was made, appropriate recruitment and one day training was given on the objective of the study, selection of study participants, how to keep confidentiality of the collected data, how to fill the data collection format and data quality management and follow-up for data collectors and supervisors. Intensive supervision was done by investigators and supervisors during the whole period of data collection.

A random sample of questionnaires were reviewed by the supervisors and the investigators to conform reliability of data before data collection and the investigators also made random cross checked for their completeness, accuracy, and consistency at the end of each day and corrective discussion was undertaken with all the research team members. Data were checked for completeness and consistency and then it was coded, entered and stored into the computer using Epi data version 3.01 statistical software. Pretest was done at Butajira town on 5% of study subjects and modification will be made accordingly. Both the validity and the reliability test were checked.

Data processing and analysis

Data were entered into Epi-data version 3.1 and exported to SPSS version 25 for Windows, then cleaned, edited, coded and exploratory data analysis was carried out to check the levels of missing values, presence of influential outliers, multi-co linearity. Crude odd ratios and AOR were computed to assess the presence of degree of association between the outcome variable and the explanatory variables.

Both bivariate and multivariable logistic regression model were fitted to assess the association between outcome and explanatory variables. Those independent variables that had p-value less than 0.25 in bivariate analysis were entered in to the multivariable logistic regressions model.

Backward stepwise regression was used for choosing determinant variables. Extent of strength was presented using odds ratios and its 95% confidence intervals. P-value ≤ 0.05 was used as a cutoff point to determine statistical significance in multiple logistic regressions for the final model. Hosmer and Lemeshow as well as omnibus test were used to test the model fitness. Multicollineaity was checked using standard error. Finally, the result was presented using texts and tables.

Ethical consideration

Ethical clearance was obtained from Wolkite University College of medicine and health sciences ethical review board and permission letter was obtained from the Guraghe Zone health department and the corresponding Woreda health care administrative offices. A written consent was obtained directly from the respondents. All the necessary measures were taken to maintain and assure the confidentiality and all benefits of the patients.

Results

The socio-demographic characteristics of the respondents

The minimum and maximum age of the respondents was 18 years old and 75 years old respectively, with the mean of 33.72±11.58 years. Nearly, half (49.4%) of the respondents had age between 36 to 64 years old. In this study, the number of males and females were almost equal, 50.5% and 49.5% were males and females respectively. Regarding the marital status, more than half (57.1%) of the respondents were married and maximum of the respondents (42.9%) were studied college and above.

Among the urban residents (142), only 18(12.7%) were applied the basic preventive measures. In addition, these basic preventive measures were applied by one fourth (19.1%) of the rural residents. Among respondents who studied college and above (272), these preventive measures were applied only by 41 (15.1%) and around three fourths (74.4%) of those respondents who studied grade 1-8 did not apply these preventive measures.

Regarding the marital status of the respondent, 241(38.0%), 362(57.1%), 13(2.1%) and 18(2.8%) of the respondents were single, married, divorced and widowed respectively. Around one fourths

(25.2%) of the respondents were students and nearly half (49.4%) of the respondents have 4-6 family members within the household. More than on fifth (21.9%) of the students and around one fourth (24.3%) of the currently not employed study subjects were applied the basic preventive measures. Regarding mass media, 526 (83.0%) of the respondents have mass media. Among those who have mass media (526), 445 (84.6%) of the respondents did not apply the basic preventive measures (**Table 1**).

The knowledge status of the respondents

In this study, 139(21.9%; 95%CI: 18.90, 25.10) of the respondents have good knowledge. Around 95% (606) of the respondents were aware of the main clinical symptoms of COVID-19. Regarding the knowledge towards high risk and prognosis, only 246(38.8%) of the respondents know that those who were elder, those with chronic illness and obese patients were commonly prone to sever cases. Similarly, 541(85.3%) of the respondents were aware about its transmission through respiratory droplets of infected individuals.

In addition, 594(93.7%) and 601(94.8%) of the respondents were know about wearing face mask and not touching eyes, nose and mouse as a preventive measures of the pandemic COVID-19. The basic preventive measures were applied on 12(8.6%) and 100(20.2%) of the respondents who have poor and good knowledge respectively. All of the respondents have information towards the pandemic COVID-19 preventive measures. Among them, 526 (83.0%), 83 (13.1%) and 25 (3.9%) of them used mass media, health care workers and neighbors as basic source respectively (**Table 2**).

Attitude status of the respondents

The mean attitude score point was 39.03 points, ranging from 19 to 50 points. The majority of the participants 597(94.2%; 95%CI: 92.30, 95.90) had a favorable attitude towards the applicability of basic preventive measures of the pandemic COVID-19. More than half (52.5%) of the participants agreed positively with all attitude questions, that stated about the prevention of the pandemic COVID-19(**Table 3**).

Comorbid health problems

Nearly, one fifths (21.8%) of the respondents had comorbid health problems. Among them, 56(8.8%) of them had DM, 53(8.4%) had hypertension, 16(2.5%) had HIV/AIDS and the remaining 13(2.1%) had both hypertension and DM.

The applicability of basic preventive measures

Among those respondents, 91(14.4%) of the respondents were used lockdown as a preventive measures of the pandemic COVID-19. In addition; 21(3.31%) of the respondents did not apply lockdown but reasonable exit with the appropriate preventive measures of COVID-19. Therefore; the magnitude of respondents who apply the basic preventive measures to the pandemic COVID-19 was 17.70% (95% CI: 14.70, 20.50) (**Table 4**). The most common mentioned reason for fail to apply the basic preventive measures of the pandemic COVID-19 were poor knowledge, poor attitude and unavailability of the preventive measures (mask and hand sanitizers due to poor finance), which was 139 (78.1%), 37(5.8%) and 147 (23.2%) respectively.

Factors associated with the applicability basic preventive measures

This study assesses factors associated with the applicability of basic preventive measures of the pandemic COVID-19. In Bivariate analysis, residence, educational level, occupational status, marital status, family size, diagnosed medical health problem and knowledge status were the candidate variables for multivariable logistic regression model. In multivariable logistic regression model, residence, educational level, occupational status, marital status, family size, diagnosed medical health problem and knowledge towards the preventive measures were statistically significant factors associated with the applicability of the pandemic COVID-19 **Table 5**).

The odd of the likelihood of not applying the basic preventive measures of the pandemic COVID-19 among rural residents were 4 times higher as compared with the urban residents (AOR: 4.78; 95%CI: 0.25, 0.89).Respondents who studied grade 1-8 were 3 times more likely not to apply the basic preventive measures as compared with those who studied college and above (AOR: 3.7; 95%CI: 1.7, 7.9). The odd of the likelihood of not applying the basic preventive measures among farmers was 4 times higher as compared with the private business employees (AOR: 4.1; 95%CI:

1.25, 13.35). Individuals currently not married were 2 times more likely not to apply the basic preventive measures as compared with currently married (AOR: 2.2; 95%CI: 1.24, 4.06).

The odd of the likelihood of the applicability of the basic preventive measures among respondents who have a family size 1-3 is 6 times higher as compared with those who have more than six families (AOR: 6.5; 95%CI: 3.21, 13.35). Similarly, residents who have a family size 4-6 were 2 times more; likely to not to apply the basic preventive measures of the pandemic COVID-19. Respondents who did not have any diagnosed medical health problems were 6 times more likely not to apply the basic preventive measures of the pandemic COVID-19 (AOR: 6.4; 95%CI: 3.85, 10.83) (**Table 6**).

Discussion

This study assesses the proportion of the respondents who apply the basic preventive measures of the pandemic COVID-19 and associated factors among the residents of Guraghe Zone. Considering the fact that Ethiopia is a multi-ethnic country with vastly different economic source, education levels, traditions, it is expected that the levels of knowledge, attitude, and applicability of basic preventive measures will also markedly differ in the population.

The proportion of residents who apply the basic preventive measures to the pandemic COVID-19 was 17.70% (95% CI: 14.70, 20.50). This study finding was less than the study conducted at Nepal (78.9%), Addis Zemen Hospital, Northwest Ethiopia (52.5%) and Northwest Gonder, Ethiopia (59.4%) [14, 15, 16]. This might be due to the difference in the socio economic status of the countries. In low income countries, the individuals did not apply the lockdown to sustain their daily lives and they cannot invest any cost in purchasing the personal protective devices [17].

The magnitude of residents who have good knowledge was 21.9%. This study finding is less than the study conducted at Nepal, Uganda and Henan China; which was 76%, 69% and 89% respectively [9, 14, 18]. This discrepancy in knowledge level may be related to the better preparedness for the worst and the number of residents who were severely affected with the infection especially in Henan China.

The proportion of individuals who have favorable attitude towards COVID-19 prevention methods was 94.2%. This study finding was greater than the study conducted at Nepal was 54.7% [14] and which was less than the study conducted at China (98%) [10]. These discrepancies might be due to the residents' knowledge level, level of health information dissemination, government implementation policies and the residents' adherence towards the prevention methods.

Consistent with the study conducted in Bangladesh [19], the odd of the likelihood of not applying the basic preventive measures of the pandemic COVID-19 among rural residents were 4 times higher as compared with the urban residents (AOR: 4.78; 95%CI: 0.25, 0.89). This might be probably due to the inaccessibility of the social and mass media especially television across the households, where media plays an important protective role through raising the public awareness about protective measures and through the countering rumors [20].

In addition; the urban populations were more prone to the information disseminations regarding the preventive measures of the pandemic COVID-19. In addition, the rural residents are prone to the cultural prejudices as compared with the urban residents [21]. Information sources are more available and spread faster in cities, and people can obtain first-hand information quickly as compared with the rural residents [22].

Inconsistent with the study conducted at Nepal and Bangladesh [14, 19], respondents who studied grade 1-8 were 3 times more likely not to apply the basic preventive measures as compared with those who studied college and above (AOR: 3.7; 95%CI: 1.7, 7.9). This is commonly due to lower grade studies were prone to lower knowledge and a weak learning ability, making it harder to grasp the relevant knowledge regarding COVID-19, unable to adopt a protective attitude and be less positive. It has been suggested that health education should be targeted at people with different educational levels and different needs for health education. For the less educated population, easy-to-understand publicity materials may be more effective.

Occupational status of the respondents became one of the factors which affect the applicability of the basic preventive measures of the pandemic COVID-19. The odd of the likelihood of fail applying the basic preventive measures among farmers was 4 times higher as compared with the private business employees (AOR: 4.1; 95%CI: 1.25, 13.35). Farmers did not adopt

recommended measures to promote safe and consistent use of preventive measures, lock down to sustain their life and did not use face mask (which became uncomfortable during farming) [23].

Individuals currently not married were 2 times more likely not to apply the basic preventive measures as compared with currently married (AOR: 2.2; 95%CI: 1.24, 4.06). This might be due to the reason that, single individuals are less likely to have family support and who did not be able to stay at home to win their daily lives as compared with the married one.

The odd of the likelihood of the applicability of the basic preventive measures among respondents who have a family size 1-3 is 6 times higher as compared with those who have more than six families (AOR: 6.5; 95%CI: 3.21, 13.35) and residents who have a family size 4-6 were 2 times more; likely to fail to apply the basic preventive measures of the pandemic COVID-19 as compared with those who have more than six families (AOR: 2.4; 95%CI: 1.35, 4.25). This might be due to that having increased number of family members have better encouragement and awareness acquired through different sources. Information missed through one member can be acquired through the other member, which can be an input for the applicability of the preventive measures of the pandemic COVID-19.

Respondents who did not have any diagnosed medical health problems were 6 times more likely fail to apply the basic preventive measures of the pandemic COVID-19 (AOR: 6.4; 95%CI: 3.85, 10.83). Patients who have an experience of infection of chronic diseases have more compliance with preventive behaviors and are more careful in the prevention of diseases [20]. In addition, it might be due to that, individuals who have chronic diseases have information about its feature of being serious illness on those who have a chronic diseases and elder populations.

Knowledge became one of the associated factors for the applicability of the basic prevention measures of the pandemic COVID-19. Individuals who have poor knowledge were 3 times more likely to fail to apply the basic preventive measures of the pandemic COVID-19 (AOR: 3.5; 95%CI: 1.60, 7.40). This is due to knowledgeable individuals are more careful and responsible for the application of the preventive measures. In addition, knowledgeable individuals know the way of prevention, mode of transmission and the risk if it was not prevented appropriately.

Conclusion

Despite the application of preventive measures and vaccine delivery, the applicability of the pandemic COVID-19 preventive measures was too low, which indicate that the Zone is at risk for the infection. Rural residents, those who have lower educational level, farmers, non-marrieds, those who have lower family size, those who have diagnosed medical illnesses and those who have poor knowledge were prone to the infection with the pandemic COVID-19 due to the lower practice of applying the basic preventive measures. In addition, awareness creation should be in practice at all levels of the community especially lower educational classes and rural residents.

Recommendations

- Awareness creation should be in practice at for all the community especially lower educational classes.
- Individuals who have lower family size should be highly followed and a follow up link should be created between them and health extension practitioners and health facilities.
- Especial emphasis should be given for the rural residents to increase their practice level.
- Should supply the basic preventive measures such as mask and sanitizers for financially poor individuals
- Should strictly follow and encourage the rural residents.
- Must work more than the usual to increase the awareness of the residents.
- Residents must practice the information gained through different sources.
- Individuals who have no diagnosed medical illnesses should apply the basic preventive measures.

Acronyms and abbreviations

AOR: Adjusted odd ratio

CDC: Center for disease control

CI: Confidence interval

COR: Crude odd ratio

COVID-19: Corona Virus Disease 2019

FMOH: Federal Ministry of health

SARS-CoV-2: Severe acute respiratory syndrome coronavirus 2

SNNPR: South Nation Nationalities and Peoples Regional state

SPSS: Statistical package for social science

RNA: Ribo nucleic acid

WHO: World Health Organization

Declarations

Consent for publication

N/A

Availability of data and materials

All relevant data are within the manuscript and its Supporting Information files.

Competing interest

I declare that the authors have no any conflict of interest.

Funding

Not applicable

Author's contribution

Samuel Dessu and Tadesse Tsehay were involved in the conceptualization, data curation, and formal analysis, funding acquisition, investigation, methodology. Tadele Girum, Abebe Timerga, Baye Tsegaye, Mamo Solomon and Abdurezak Kemal were involved in project administration, resource, software, validation. Mulugeta Geremew, Biru Migora, Yibeltal Mesfin, Fisha Alebel were involved in visualization and writing the original draft. Omega Tolosa, Shegaw Tesfa and Fedila Yasin were involved in the supervision, writing - review and editing. All authors reviewed the manuscript.

Author's information

Samuel Dessu: Department of Public Health, College of Medicine and health Sciences, Wolkite University, Wolkite, Ethiopia**.** Tadesse Tsehay: Department of Nursing, College of Medicine and health Sciences, Wolkite University, Wolkite, Ethiopia**.** Tadele Girum: Department of Public Health, College of Medicine and health Sciences, Wolkite University, Wolkite, Ethiopia**.** Abebe Timerga: Department of Biomedical Sciences, College of Medicine and health Sciences, Wolkite University, Wolkite, Ethiopia**.** Mamo Solomon: Department of Nursing, College of Medicine and health Sciences, Wolkite University, Wolkite, Ethiopia**.** Baye Tsegaye: Department of Nursing, College of Medicine and health Sciences, Wolkite University, Wolkite, Ethiopia**.** Mulugeta Geremew: Department of Statistics, College of Natural and Computational Sciences, Wolkite University, Wolkite, Ethiopia**.** Biru Migora: Department of Statistics, College of Natural and Computational Sciences, Wolkite University, Wolkite, Ethiopia**.** Yibeltal Mesfin: Department of Midwifery, College of Medicine and health Sciences, Wolkite University, Wolkite, Ethiopia**.** Abdurezak Kemal: Department of Public Health, College of Medicine and health Sciences, Wolkite University, Wolkite, Ethiopia**.** Fisha Alebel: Department of Nursing, College of Medicine and health Sciences, Wolkite University, Wolkite, Ethiopia**.** Omega Tolosa: Department of Nursing, College of Medicine and health Sciences, Wolkite University, Wolkite, Ethiopia**.** Shegaw Tesfa: Department of Nursing, College of Medicine and health Sciences, Wolkite University, Wolkite, Ethiopia

Acknowledgements

We would like to thank the mothers who are directly involved in the study and administrator of each hospital for their effort and permission to conduct the study.

Corresponding Author

Correspondence to Samuel Dessu

References

1. Zhu, H., Wei, L. & Niu, P. The novel coronavirus outbreak in Wuhan, China. glob health res policy 5, 6 (2020).
2. World Health organization: Coronavirus disease 2019 (COVID-19) Situation Report – 94; 2020.
3. Ebrahim SH, Ahmed QA, Gozzer E, Schlagenhauf P, Memish ZA. Covid-19 and community mitigation strategies in a pandemic. BMJ. 2020;368.
4. UNICEF: for every child: Everything you need to know about washing your hands to protect against coronavirus (COVID-19); 2020.
5. Roba HS, Beyene AS, Mengesha MM, Ayele BH. Prevalence of Hypertension and Associated Factors in Dire Dawa City, Eastern Ethiopia: A Community-Based Cross-Sectional Study. International Journal of Hypertension. 2019;2019:9878437.

6. Wang C, Pan R, Wan X, Tan Y, Xu L, Ho CS. Immediate Psychological Responses and Associated Factors during the Initial Stage of the 2019 Coronavirus Disease (COVID-19) Epidemic among the General Population in China. Int J Environ Res Public Health. 2020;17(1729).
7. Huang C, Wang Y, Li X, Ren L, Zhao J, Hu Y, et al. Clinical features of patients infected with 2019 novel coronavirus in Wuhan, China. The Lancet. 2020;395:497-506.
8. Varshney M, Parel JT, Raizada N, Sarin SK. Initial psychological impact of COVID-19 and its correlates in Indian Community: An online (FEEL-COVID) survey. PLOS ONE. 2020;15(5):1-10.
9. M Z, Zhou M, Tang F, Wang Y, Nie H, Zhang L, et al. Knowledge, attitude, and practice regarding COVID-19 among healthcare workers in Henan, China. J Hosp Infect. 2020;105:183-7.
10. Gao H, Rujun H, Ling Y, Xiaoli Y, Hao T, Lan L, et al. Knowledge, attitudes and practices of the Chinese public with respect to coronavirus disease (COVID-19): an online crosssectional survey. BMC Public Health. 2020;20(1816).
11. World Health Organization: Ethiopia. 2.2 million COVID-19 vaccines allocated by the COVAX Facility arrive in Ethiopia, marking the start of the country's COVID-19 vaccination campaign: 2021.
12. Kebede Y, Yitayih Y, Birhanu Z, Mekonen S, Ambelu A. Knowledge, perceptions and preventive practices towards COVID-19 early in the outbreak among Jimma university medical center visitors, Southwest Ethiopia. PLOS ONE. 2020;15(5).
13. Cirrincione L, Plescia F, Ledda C, Rapisarda V, Martorana D, Moldovan RE, et al. COVID-19 Pandemic: Prevention and Protection Measures to Be Adopted at the Workplace. Sustainability. 2020;12:3603.
14. Nira T, Punam R, Siddhartha D, Binod S, Bikash S, Prajjwal P, et al. COVID-19: a National Survey on perceived level of knowledge, attitude and practice among frontline healthcare Workers in Nepal. BMC Public Health. 2020;20(1905).
15. Akalu Y, Ayelign B, Molla MD. Knowledge, Attitude and Practice Towards COVID-19 Among Chronic Disease Patients at Addis Zemen Hospital, Northwest Ethiopia. Infection and Drug Resistance. 2020;13:1949—60.
16. Shibabaw T, Teferi B. Knowledge and Practice Toward Prevention of SARS-CoV-2 Among Healthcare Workers at Delghi Primary Hospital During a Massive Test Campaign in Northwest Gondar, Ethiopia: Institution-Based Descriptive Cross-Sectional Survey. Infection and Drug Resistance. 2021;14:381—90.
17. Mulu GB, Kebede WM, Worku SA, Mittiku YM, Ayelign B. Preparedness and Responses of Healthcare Providers to Combat the Spread of COVID-19 Among North Shewa Zone Hospitals, Amhara, Ethiopia, 2020. Infection and Drug Resistance. 2020;13:3171-8.
18. Olum R, Chekwech G, Wekha G, Nassozi DR, Bongomin F. Coronavirus Disease-2019: Knowledge, Attitude, and Practices of Health Care Workers at Makerere University Teaching Hospitals, Uganda. Front Public Health. 2020;8(181).
19. Ferdous MZ, Islam MS, Sikder MT, Mosaddek ASM, Zegarra-Valdivia JA, Gozal D. Knowledge, attitude, and practice regarding COVID-19 outbreak in Bangladesh: An onlinebased cross-sectional study. PLoS ONE. 2020;15(10):e0239254.

20. Zeinab AK, Wael AB, Shimaa KZ, Mohammed GH, Safa HA, Enas Z, et al. Knowledge, Attitude and Practice toward COVID-19 among Egyptians. Journal of Epidemiology and Global Health. 2020;10(4).
21. Feng J, Li TG, Qin PZ, Lu JY, Chen C, Chen ZQ, et al. Telephone survey and analysis of flu among citizens in Guangzhou. S China J Prev Med. 2013;39(4):35-7.
22. Xu YF, Li P, Zhou J, Guo LF. Investigation on cognitive behavior and psychological status of SARS in residents of Nanning city. Pract Prev Med. 2005;12(1):134-5.
23. Bonnie JM, Catherine BH. Health hazaeds and preventive measures of farm women. AAOHN Journal. 2002;50(7).

Table legends

Table 1: The Socio-demographic characteristics of residents in Guraghe Zone, 2020 (n=634)

Table 2: Knowledge status of the respondents on COVID-19 among residents in Guraghe Zone, 2020 (n=634)

Table 3 : The level of attitude towards the prevention of COVID-19 among residents in Guraghe Zone, 2020 (n=634)

Table 4: The applicability of the basic preventive measures of the pandemic COVID-19 among residents in Guraghe Zone, 2020 (n=634)

Table 5: Bivariable analysis of factors associated with the applicability of the basic preventive measures of the pandemic COVID-19 among residents in Guraghe Zone, 2020 (n=634)

Table 6: Factors associated with the applicability of the basic preventive measures of the pandemic COVID-19 among residents in Guraghe Zone, 2020 (n=634)

Table 1:

Variables	Category	Frequency	Percent
Age (in years)	18-35	205	32.3
	36-64	313	49.4
	>64	116	18.3
Residence	Urban	142	22.4
	Rural	492	77.6
Educational status	Unable to read and write	51	8.0
	Only read and write	59	9.3
	Grade 1-8	121	19.1
	Grade 9-12	131	20.7
	College and above	272	42.9
Occupational status	Farmer	83	13.1
	Student	160	25.2
	Currently not employed	37	5.8
	Government employed	248	39.1
	Private business employed	106	16.7
Religious status	Orthodox	378	59.6
	Muslim	162	25.6
	Protestant	60	9.5
	Catholic	26	4.1
	Others	8	1.3
Family size	1-3	205	32.3
	4-6	313	49.4
	>6	116	18.3

Table 2

Knowledge assessment questions	Knowledge Category		
	Yes	No	I don't know
**Knowledge of cause and symptoms**			
The main clinical symptoms of COVID-19 are fever, fatigue, dry cough, and myalgia	606(95.6%)	14(2.2%)	14(2.2%)
Unlike the common cold, stuffy nose, runny nose, and sneezing are less common in persons infected with the COVID-19 virus	261(41.2%)	332(52.4%)	41(6.5%)
**Knowledge of high risk and prognosis**			
Not all persons with COVID-19 will develop severe cases. Only those who are elderly, have chronic illnesses & are obese are more likely to be severe cases	246(38.8%)	350(55.2%)	38(6.0%)
There currently is no effective cure for COVID-19, but early symptomatic and supportive treatment can help most patients recover from the infection	188(29.7%)	437(68.9%)	9(1.4%)
**Knowledge about mode of transmission and infectiousness**			
The COVID-19 virus spreads via respiratory droplets of infected individuals	541(85.3%)	82(12.9%)	11(1.7%)
Eating or contacting wild animals would result in the infection by the COVID-19 virus	416(65.6%)	154(24.3%)	64(10.1%)
Persons with COVID-19 cannot infect the virus to others when a fever is not present	112(17.7%)	489(77.1%)	33(5.2%)
**Knowledge about ways of prevention**			
Proper washing hand with soap and water is one method of preventing COVID-19	594(93.7%)	40(6.3%)	0(0%)
One way of prevention of COVID-19 is not touching the eye, nose by unwashed hands	601(94.8%)	33(5.2%)	0(0%)
To prevent the infection by COVID-19, individuals should	542(85.5%)	89(14.0%)	3(0.5%)

avoid going to crowded places such as train stations, market and avoid taking public transportations			
Ordinary residents can wear general medical masks to prevent the infection by the COVID- 19 virus	593(93.5%)	35(5.5%)	6(0.9%)
People who have contact with someone infected with the COVID-19 virus should be immediately isolated in a proper place	563(88.8%)	69(10.9%)	2(0.3%)
Isolation and treatment of people who are infected with the COVID-19 virus are effective ways to reduce the spread of the virus	596(94.0%)	34(5.4%)	5.4%)
Children and young adults don’t need to take measures to prevent the infection by the COVID-19 virus	106(16.7%)	501(79.0%)	27(4.3%)

Table 3

Attitude domain	Level of attitude				
	Strongly disagree	Disagree	Neutral	Agree	Strongly agree
Do you agree that the Ethiopian government was handling the COVID-19 health crisis well?	52(8.2%)	126(19.9%)	37(5.8%)	364(57.4%)	55(8.7%)
Do you agree that COVID-19 will be successfully controlled?	37(5.8%)	87(13.7%)	65(10.3%)	391(61.7%)	54(8.5%)
Do you agree that Ethiopian would be able to win the battle against COVID-19?	24(3.8%)	38(6.0%)	31(4.9%)	425(67.0%)	116(18.3 %)
Do you agree that Self-protection is necessary for the protection of others?	8(1.3%)	18(2.8%)	12(1.9%)	402(63.4%)	194(30.6 %)
Do you agree that Lockdown is an effective measure to control the transmission?	27(4.3%)	59(9.3%)	20(3.2%)	334(52.7%)	194(30.6 %)
Do you agree that wearing face mask protects against COVID-19?	3(0.5%)	31(4.9%)	5(0.8%)	421(66.4%)	174(27.4 %)
Do you agree that appropriate hand washing practice is important in the prevention of COVID-19?	5(0.8%)	17(2.7%)	6(0.9%)	409(64.5%)	197(31.1 %)
Do you agree that the transmission of COVID-19 can be minimized in keeping physical distance?	17(2.7%)	37(5.8%)	14(2.2%)	386(60.9%)	180(28.4 %)
Did you agree that COVID-19 leads to stigma?	45(7.1%)	75(11.8%)	21(3.3%)	369(58.2%)	124(19.6 %)
Do you agree that if getting COVID-19, you will accept isolation in	30(4.7%)	67(10.6%)	14(2.2%)	344(54.3%)	179(28.2

health facilities?					%)

Table 4

**Variables**	**Applicability of preventive measures**	
	**Yes**	**No**
Lockdown	91(14.4%)	543(85.6%)
Appropriate frequent hand washing	253(39.9%)	381(60.1%)
Stop hand shaking	152(24.0%)	482(76.0%)
Avoid touching eye, nose, and mouth	110(17.4%)	524(82.6%)
Using face mask as a preventive measure	88(13.9%)	546(86.1%)
Apply two meters physical distance	63(9.9%)	571(90.1%)
Avoided going to the crowed place	79(12.5%)	555(87.5%)
Using elbow to cover during coughing/sneezing	454(71.6%)	180(28.4%)

Table 5

Variables	Category	Status		COR (95%CI)
		Not yet applied basic preventive measures	Applied basic preventive measures	
Age (in years)	18-35	349	68	2.2(0.55, 8.72)
	36-64	166	41	1.7(0.43, 7.00)
	>64	7	3	1
Sex	Male	267	53	1
	Female	255	59	0.85(0.57, 1.29)
Residence	Urban	124	18	1
	Rural	398	94	0.62(0.36, 1.06)*
Educational level	Unable to read and write	47	4	2.08(0.71, 6.10)*
	Only read and write	52	7	1.32(0.56, 3.10)*
	Grade 1-8	90	31	0.51(0.30, 0.87)*
	Grade 9-12	102	29	0.62(0.37, 1.06)*
	College and above	231	41	1
Occupational status	Farmer	78	5	3.62(1.29, 10.13)*
	Student	125	35	0.83(0.45, 1.54)
	Currently not employed	28	9	0.72(0.29, 1.77)
	Government employed	205	43	1.2(0.62, 1.99)
	Private business employed	86	20	1
Marital status	Currently married	284	78	1
	Currently not married	238	34	1.9(1.24, 2.98)*
Family size	1-3	188	17	6.0(3.23, 11.30)*
	4-6	259	54	2.62(1.62, 4..24)*
	>6	75	41	1
Mass media availability	Yes	445	81	1
	No	77	31	0.45(0.28, 0.73)*
Diagnosed medical health problem	Yes	82	56	1
	No	440	56	5.36(3.46, 8.32)*

Knowledge status	Good	127	12	1
	Poor	395	100	0.37(1.19, 0.70)*
Attitude	Favorable	33	4	1
	Unfavorable	489	108	0.55(0.19, 1.58)
*Key note: *indicates variables which have p-value <0.25 in bivariable analysis*				

Table 6

Variables	Category	COR (95%CI)	AOR(95%CI)	P-value
Residence	Urban	1	1	0.0047
	Rural	0.62(0.36, 1.06)*	4.78(2.5, 8.9)**	
Educational level	Unable to read and write	2.08(0.71, 6.10)*	1.03(0.28, 3.88)	0.011
	Only read and write	1.32(0.56, 3.10)*	0.42(0.13, 1.37)	
	Grade 1-8	0.51(0.30, 0.87)*	3.7(1.70, 7.90)**	
	Grade 9-12	0.62(0.37, 1.06)*	0.55(0.27, 1.14)	
	College and above	1	1	
Occupational status	Farmer	3.62(1.29, 10.13)*	4.1(1.25, 13.35)**	0.027
	Student	0.83(0.45, 1.54)	0.45(0.21, 1.01)	
	Currently not employed	0.72(0.29, 1.77)	0.57(0.0.19, 1.72)	
	Government employed	1.2(0.62, 1.99)	0.47(0.19, 1.01)	
	Private business employed	1	1	
Marital status	Currently married	1	1	0.003
	Currently not married	1.9(1.24, 2.98)*	2.2(1.24, 4.06)**	
Family size	1-3	6.0(3.23, 11.30)*	6.5(3.21, 13.35)**	0.0001
	4-6	2.62(1.62, 4..24)*	2.4(1.35, 4.25)**	
	>6	1	1	
Diagnosed medical health problem	Yes	1	1	0.0001
	No	5.36(3.46, 8.32)*	6.4(3.85, 10.83)**	
Knowledge status	Good	1	1	0.002
	Poor	0.37(1.19, 0.70)*	3.5(1.60, 7.40)	

*Key note: *indicates variables which have p-value <0.25, **indicates variables which have p-value <0.05*

Click here to access/download
Supporting Information
COVID SPSS data.sav

The applicability of basic preventive measures of the pandemic COVID-19 and associated factors among residents in Guraghe Zone

Samuel Dessu[1]*, Tadesse Tsehay[2], Tadele Girum[1], Abebe Timerga[3], Mamo Solomon[2], Baye Tsegaye[2], Mulugeta Geremew[4], Biru Migora[4], Yibeltal Mesfin[5], Abdurezak Kemal[1], Fisha Alebel[2], Omega Tolosa[2], Shegaw Tesfa[2], Fedila Yasin[1]

[1]Department of Public Health, College of Medicine and Health Sciences, Wolkite University, Wolkite, Ethiopia
[2]Department of Nursing, College of Medicine and health Sciences, Wolkite University, Wolkite, Ethiopia
[3]Department of Biomedical Sciences, College of Medicine and health Sciences, Wolkite University, Wolkite, Ethiopia
[4]Department of Statistics, College of Natural and Computational Sciences, Wolkite University, Wolkite, Ethiopia
[5]Department of Midwifery, College of Medicine and health Sciences, Wolkite University, Wolkite, Ethiopia

Correspondence to Samuel Dessu [Email: dessusamuel@yahoo.com, Tel: +251910883594] & Tadesse Tsehay [Email: tadesse200912@gmail.com, Tel: +251961818585]

Abstract

Introduction: Internationally, countries have reacted to the COVID-19 outbreak by introducing key public health non-pharmaceutical interventions to protect vulnerable population groups. In response to COVID-19, the Government of Ethiopia has been taking a series of policy actions beyond public health initiatives alone. Therefore, this study was aimed to assess the applicability of basic preventive measures of the pandemic COVID-19 and associated factors among the residents of Guraghe Zone from 18th to 29th September, 2020.

Methods: Community based cross sectional study was conducted at Guraghe Zone from 18th to 29th September, 2020. Systematic random sampling method was applied among the predetermined 634 samples. Variables which had p-value less than 0.25 in bivariate analysis were considered as candidate for multivariable logistic regression model. P-value $\leq$ 0.05 was used as a cutoff point to determine statistical significance in multiple logistic regressions for the final model.

Result: In this study, 17.70% (95% CI: 14.70, 20.50) of the respondents apply the basic preventive measures towards the prevention of the pandemic COVID-19. In addition, being rural resident (AOR: 4.78,; 95%CI: 2.5, 8.9), being studied grade 1-8 (AOR: 3.7; 95%CI: 1.7, 7.9), being a farmer (AOR: 4.1; 95%CI: 1.25, 13.35), currently not married (AOR: 2.2, 95%CI: 1.24, 4.06), having family size 1-3(AOR: 6.5; 95%CI: 3.21, 3.35), have no diagnosed medical illness (AOR: 6.4; 95%CI: 3.85, 10.83) and having poor knowledge (AOR: 3.5; 95%CI: 1.60, 7.40) were factors which are statistically significant in multivariable logistic regression model.

Conclusion: Despite the application of preventive measures and vaccine delivery, the applicability of the pandemic COVID-19 preventive measures was too low, which indicate that the Zone is at risk for the infection. Rural residents, those who have lower educational level, farmers, non-marrieds, those who have lower family size, those who have diagnosed medical illnesses and those who have poor knowledge were prone to the infection with the pandemic COVID-19 due to the lower practice of applying the basic preventive measures. In addition, awareness creation should be in practice at all levels of the community especially lower educational classes and rural residents.

Key words: COVID-19; Preventive measures; Applicability; Guraghe Zone; Pandemic

Introduction

The pandemic of coronavirus disease 2019 (COVID-19) started in December 2019 in Wuhan, China [1]. Currently, the virus causing the disease is approaches each continent throughout the world and continues to spread at an alarming rate [2].

Globally, countries have acted up on the COVID-19 outbreak through introducing key public health non-pharmaceutical interventions to protect vulnerable population groups [3]. Lock down, washing hands frequently using soap in mashing for more than 20 seconds, maintain physical distancing, stay informed and follow advice given by healthcare professionals and seeking medical advice if develop cough or fever or experience difficulty of breathing and call in advance the center assigned for COVID-19 response were the recommended measures to reduce the transmission of COVID-19 by world health Organization (WHO) [4].

COVID-19 can results in a severe national problems. In addition; it results in severe psychological, economic crisis across the globe [5, 6, 7, 8]. As the disease progressed, concerns regarding health, economy, and livelihood increased day-to-day. The findings of the pandemic's impact on these issues could help inform health officials and the public to provide mental health interventions to those who are in need [8].

The first case of COVID-19 in Ethiopia was detected on 13th March 2020 involving two tourists from Japan [1]. In accordance with the applied case definition and testing strategies, till 8th of April, 2021, the number of peoples infected with the pandemic COVID-19 in the world was 132,730,691, of them, 3,148, 358 were in Africa and of them, 221,544 were in Ethiopia. Among them, globally 2,880,726 were died, of them, 114,610 were in Africa and of them, 3,058 were in Ethiopia [4].

In response to COVID-19, the Government of Ethiopia has been taking a series of policy actions beyond public health initiatives alone [9]. These include closing schools, restricting use of public transportation, banning large meetings, and suspending sporting and religious gatherings. A state of emergency has been put in effect and staying at home and working from there has been strongly advised [10]. On March 2021, Ethiopia has received 2.184 million doses of the Astra Zeneca COVID-19 vaccine via the COVAX facility and the country is on immunizing the frontline workers and the most susceptible individuals [11].

Despite the implementation of such strategies, the occurrence of the pandemic COVID-19 is still increasing. Therefore, this study focus on the evaluation of the applicability of basic prevention methods of the pandemic COVID-19 may help to the populations at risk to reduce the risk of mortality.

Methods and materials

Study Design, Area and Period

A community based cross-sectional study was conducted at Guraghe Zone from 18th to 29th September, 2020. Guraghe Zone is one of the 13 Zones encountered at South Nations Nationalities and Peoples regional state (SNNPR). Wolkite town is its administrative sit and located at 158 km south from Addis Ababa. Based on the 2007 Ethiopian national population and housing census, the population of the Zone is projected to be about 1,609,908 and 51.38% are females. Administratively the Zone is divided in to 16 districts (Woredas) and 4 city administrations.

Populations

Populations

In this study, all the residents at the Guraghe Zone were considered as a source population and all the selected populations in the Guraghe Zone from 18th to 29th September, 2020 from the source population were termed as study populations.

Inclusion and exclusion criteria

All the ambulatory residents of the Guraghe Zone were included into the study and residents of Guraghe Zone who cannot able to communicate (unable to listen and talk) and children who have age less than 15 were excluded from the study.

Sample size determination and sampling procedures

The required sample size was determined using formula for single population proportion formula n = $\frac{z_{\frac{\alpha}{2}}^{2}P(1-P)}{d^2}$, Where n denotes the sample size, $z_{\frac{\alpha}{2}}$ denotes the reliability coefficient of standard

error, at 5% level of significance (which is 1.96), P = proportion of residents applicability of basic preventive measures at Guraghe zone (Which is 50% because there was no study conducted before) and d = margin of error. Therefore, the calculated sample size was 384. Using design effect for the sample in using interclass correlation (δ) = 1.5, it became 576. Through considering 10% for non-response rate, the final sample size for the study was 634.

Among the 20 districts found in Guraghe Zone, 7 districts (Abeshge Woreda, Emdebir town administration, Enemor Woreda, Edja woreda, Gumer Woreda, Meskan woreda and Wolkite town administration) were randomly selected using lottery method. From town administrations, four sub cities (two sub cities from Emdebir town administration and two sub cities from Wolkite town administration) were randomly selected using lottery method. Again from the randomly selected rural districts, 23 kebelles were randomly selected (Three kebelles from Abeshge Woreda, five Kebelles from Enemor Woreda, Four Kebelles from Edja Woreda, Five Kebelles from Gumer woreda and six kebelles from Meskan woreda).

Finally, the calculated sample size was proportionally allocated to the randomly selected districts (Abeshge (28), Emdeber (31), Enemor (156), Edja (121), Gumer (114), Meskan (137) and Wolkite town administration (47)) and the households from each district were selected using systematic random sampling method. Within each of the selected household, one individual was taken, primarily the house hold head (father) or the mother or kids based on the hierarchy (eldest to youngest).

Study variables

The dependent variable for this study was the applicability of basic COVID-19 preventive measures and the independent variables were socio demographic variables (age, sex, residence, educational status, occupation, marital status, religion and estimated monthly income), knowledge status of the respondents, attitude towards COVID-19, availability of Mass media and diagnosed medical health problem (Comorbidity)

Operational definitions

High knowledge: If the respondent answers at least 11 of the 14 knowledge assessment questions correctly [12].

Moderate knowledge: If the respondent answers at least nine of the 14 knowledge assessment questions correctly [12].

Poor knowledge: If the respondent responds $\leq$ 8 knowledge assessment questions correctly [12].

Good knowledge: If the respondent responds $\geq$ 9 knowledge assessment questions correctly [12].

Proper hand washing practice: If the respondent wash his/her hands using soap and water in mashing his hands for more than 20 seconds or using hand sanitizers [13].

Favorable attitude towards COVID-19: a mean attitude score $\geq$30 [12].

Unfavorable attitude towards COVID-19: a mean attitude score less than 30 [12].

Apply basic preventive measures: if the respondent applies complete lockdown (stay at home) or reasonable exit in wearing mask appropriately, proper hand washing practice and keeps physical distance [13].

Data collection tool and procedures

Data collection tool was adapted from WHO resources and similar studies. Initially, it was prepared in English and was translated to Amharic by language experts to ensure consistency. Data were collected using structured interviewer administered questionnaires. Data were collected by 15-experienced BSC nurses and were supervised by three Msc holders in health science through the entire data collection process.

The knowledge questions have 14 items. These items include the participant knowledge about clinical presentations (items 1–4), transmission routes (items 5–8) and prevention and control (items 9–14) of COVID-19. Participants were given “true,” “false,” or “I don’t know” response options to these items. A correct response to an item were assigns 1 point, while an incorrect/don’t know response assigned 0 points. The maximum total score ranged from 0–14, with a higher score indicating better knowledge about COVID-19.

To measure attitudes towards COVID-19, 10 items (with minimum score 10 and maximum score 50) were used. Response of each item was recorded on 5-point Likert scale as follows strongly disagree (1-point), disagree (2-point), neutral (3-point), agree (4-point), and strongly agree (5-point). The overall level of attitude was categorized using original Bloom’s cut-off point, as

positive if the score was 80-100%(40-50), neutral if the score was 60-79% (30-40) and negative if the score was less than 60 (10-29). A mean score $\geq$30 was carried out as a favorable attitude and a score less than 30 indicated an unfavorable attitude toward COVID-19.

Data quality management

Data quality was assured by caring out careful design of data collection tool and appropriate modification was made, appropriate recruitment and one day training was given on the objective of the study, selection of study participants, how to keep confidentiality of the collected data, how to fill the data collection format and data quality management and follow-up for data collectors and supervisors. Intensive supervision was done by investigators and supervisors during the whole period of data collection.

A random sample of questionnaires were reviewed by the supervisors and the investigators to conform reliability of data before data collection and the investigators also made random cross checked for their completeness, accuracy, and consistency at the end of each day and corrective discussion was undertaken with all the research team members. Data were checked for completeness and consistency and then it was coded, entered and stored into the computer using Epi data version 3.01 statistical software. Pretest was done at Butajira town on 5% of study subjects and modification will be made accordingly. Both the validity and the reliability test were checked.

Data processing and analysis

Data were entered into Epi-data version 3.1 and exported to SPSS version 25 for Windows, then cleaned, edited, coded and exploratory data analysis was carried out to check the levels of missing values, presence of influential outliers, multi-co linearity. Crude odd ratios and AOR were computed to assess the presence of degree of association between the outcome variable and the explanatory variables.

Both bivariate and multivariable logistic regression model were fitted to assess the association between outcome and explanatory variables. Those independent variables that had p-value less than 0.25 in bivariate analysis were entered in to the multivariable logistic regressions model.

Backward stepwise regression was used for choosing determinant variables. Extent of strength was presented using odds ratios and its 95% confidence intervals. P-value ≤ 0.05 was used as a cutoff point to determine statistical significance in multiple logistic regressions for the final model. Hosmer and Lemeshow as well as omnibus test were used to test the model fitness. Multicollineaity was checked using standard error. Finally, the result was presented using texts and tables.

Ethical consideration

Ethical clearance was obtained from Wolkite University College of medicine and health sciences ethical review board and permission letter was obtained from the Guraghe Zone health department and the corresponding Woreda health care administrative offices. A written consent was obtained directly from the respondents. All the necessary measures were taken to maintain and assure the confidentiality and all benefits of the patients.

Results

The socio-demographic characteristics of the respondents

The minimum and maximum age of the respondents was 18 years old and 75 years old respectively, with the mean of 33.72±11.58 years. Nearly, half (49.4%) of the respondents had age between 36 to 64 years old. In this study, the number of males and females were almost equal, 50.5% and 49.5% were males and females respectively. Regarding the marital status, more than half (57.1%) of the respondents were married and maximum of the respondents (42.9%) were studied college and above.

Among the urban residents (142), only 18(12.7%) were applied the basic preventive measures. In addition, these basic preventive measures were applied by one fourth (19.1%) of the rural residents. Among respondents who studied college and above (272), these preventive measures were applied only by 41 (15.1%) and around three fourths (74.4%) of those respondents who studied grade 1-8 did not apply these preventive measures.

Regarding the marital status of the respondent, 241(38.0%), 362(57.1%), 13(2.1%) and 18(2.8%) of the respondents were single, married, divorced and widowed respectively. Around one fourths

(25.2%) of the respondents were students and nearly half (49.4%) of the respondents have 4-6 family members within the household. More than on fifth (21.9%) of the students and around one fourth (24.3%) of the currently not employed study subjects were applied the basic preventive measures. Regarding mass media, 526 (83.0%) of the respondents have mass media. Among those who have mass media (526), 445 (84.6%) of the respondents did not apply the basic preventive measures (**Table 1**).

The knowledge status of the respondents

In this study, 139(21.9%; 95%CI: 18.90, 25.10) of the respondents have good knowledge. Around 95% (606) of the respondents were aware of the main clinical symptoms of COVID-19. Regarding the knowledge towards high risk and prognosis, only 246(38.8%) of the respondents know that those who were elder, those with chronic illness and obese patients were commonly prone to sever cases. Similarly, 541(85.3%) of the respondents were aware about its transmission through respiratory droplets of infected individuals.

In addition, 594(93.7%) and 601(94.8%) of the respondents were know about wearing face mask and not touching eyes, nose and mouse as a preventive measures of the pandemic COVID-19. The basic preventive measures were applied on 12(8.6%) and 100(20.2%) of the respondents who have poor and good knowledge respectively. All of the respondents have information towards the pandemic COVID-19 preventive measures. Among them, 526 (83.0%), 83 (13.1%) and 25 (3.9%) of them used mass media, health care workers and neighbors as basic source respectively (**Table 2**).

Attitude status of the respondents

The mean attitude score point was 39.03 points, ranging from 19 to 50 points. The majority of the participants 597(94.2%; 95%CI: 92.30, 95.90) had a favorable attitude towards the applicability of basic preventive measures of the pandemic COVID-19. More than half (52.5%) of the participants agreed positively with all attitude questions, that stated about the prevention of the pandemic COVID-19(**Table 3**).

Comorbid health problems

Nearly, one fifths (21.8%) of the respondents had comorbid health problems. Among them, 56(8.8%) of them had DM, 53(8.4%) had hypertension, 16(2.5%) had HIV/AIDS and the remaining 13(2.1%) had both hypertension and DM.

The applicability of basic preventive measures

Among those respondents, 91(14.4%) of the respondents were used lockdown as a preventive measures of the pandemic COVID-19. In addition; 21(3.31%) of the respondents did not apply lockdown but reasonable exit with the appropriate preventive measures of COVID-19. Therefore; the magnitude of respondents who apply the basic preventive measures to the pandemic COVID-19 was 17.70% (95% CI: 14.70, 20.50) (**Table 4**). The most common mentioned reason for fail to apply the basic preventive measures of the pandemic COVID-19 were poor knowledge, poor attitude and unavailability of the preventive measures (mask and hand sanitizers due to poor finance), which was 139 (78.1%), 37(5.8%) and 147 (23.2%) respectively.

Factors associated with the applicability basic preventive measures

This study assesses factors associated with the applicability of basic preventive measures of the pandemic COVID-19. In Bivariate analysis, residence, educational level, occupational status, marital status, family size, diagnosed medical health problem and knowledge status were the candidate variables for multivariable logistic regression model. In multivariable logistic regression model, residence, educational level, occupational status, marital status, family size, diagnosed medical health problem and knowledge towards the preventive measures were statistically significant factors associated with the applicability of the pandemic COVID-19 **Table 5**).

The odd of the likelihood of not applying the basic preventive measures of the pandemic COVID-19 among rural residents were 4 times higher as compared with the urban residents (AOR: 4.78; 95%CI: 0.25, 0.89).Respondents who studied grade 1-8 were 3 times more likely not to apply the basic preventive measures as compared with those who studied college and above (AOR: 3.7; 95%CI: 1.7, 7.9). The odd of the likelihood of not applying the basic preventive measures among farmers was 4 times higher as compared with the private business employees (AOR: 4.1; 95%CI:

1.25, 13.35). Individuals currently not married were 2 times more likely not to apply the basic preventive measures as compared with currently married (AOR: 2.2; 95%CI: 1.24, 4.06).

The odd of the likelihood of the applicability of the basic preventive measures among respondents who have a family size 1-3 is 6 times higher as compared with those who have more than six families (AOR: 6.5; 95%CI: 3.21, 13.35). Similarly, residents who have a family size 4-6 were 2 times more; likely to not to apply the basic preventive measures of the pandemic COVID-19. Respondents who did not have any diagnosed medical health problems were 6 times more likely not to apply the basic preventive measures of the pandemic COVID-19 (AOR: 6.4; 95%CI: 3.85, 10.83) (**Table 6**).

Discussion

This study assesses the proportion of the respondents who apply the basic preventive measures of the pandemic COVID-19 and associated factors among the residents of Guraghe Zone. Considering the fact that Ethiopia is a multi-ethnic country with vastly different economic source, education levels, traditions, it is expected that the levels of knowledge, attitude, and applicability of basic preventive measures will also markedly differ in the population.

The proportion of residents who apply the basic preventive measures to the pandemic COVID-19 was 17.70% (95% CI: 14.70, 20.50). This study finding was less than the study conducted at Nepal (78.9%), Addis Zemen Hospital, Northwest Ethiopia (52.5%) and Northwest Gonder, Ethiopia (59.4%) [14, 15, 16]. This might be due to the difference in the socio economic status of the countries. In low income countries, the individuals did not apply the lockdown to sustain their daily lives and they cannot invest any cost in purchasing the personal protective devices [17].

The magnitude of residents who have good knowledge was 21.9%. This study finding is less than the study conducted at Nepal, Uganda and Henan China; which was 76%, 69% and 89% respectively [9, 14, 18]. This discrepancy in knowledge level may be related to the better preparedness for the worst and the number of residents who were severely affected with the infection especially in Henan China.

The proportion of individuals who have favorable attitude towards COVID-19 prevention methods was 94.2%. This study finding was greater than the study conducted at Nepal was 54.7% [14] and which was less than the study conducted at China (98%) [10]. These discrepancies might be due to the residents' knowledge level, level of health information dissemination, government implementation policies and the residents' adherence towards the prevention methods.

Consistent with the study conducted in Bangladesh [19], the odd of the likelihood of not applying the basic preventive measures of the pandemic COVID-19 among rural residents were 4 times higher as compared with the urban residents (AOR: 4.78; 95%CI: 0.25, 0.89). This might be probably due to the inaccessibility of the social and mass media especially television across the households, where media plays an important protective role through raising the public awareness about protective measures and through the countering rumors [20].

In addition; the urban populations were more prone to the information disseminations regarding the preventive measures of the pandemic COVID-19. In addition, the rural residents are prone to the cultural prejudices as compared with the urban residents [21]. Information sources are more available and spread faster in cities, and people can obtain first-hand information quickly as compared with the rural residents [22].

Inconsistent with the study conducted at Nepal and Bangladesh [14, 19], respondents who studied grade 1-8 were 3 times more likely not to apply the basic preventive measures as compared with those who studied college and above (AOR: 3.7; 95%CI: 1.7, 7.9). This is commonly due to lower grade studies were prone to lower knowledge and a weak learning ability, making it harder to grasp the relevant knowledge regarding COVID-19, unable to adopt a protective attitude and be less positive. It has been suggested that health education should be targeted at people with different educational levels and different needs for health education. For the less educated population, easy-to-understand publicity materials may be more effective.

Occupational status of the respondents became one of the factors which affect the applicability of the basic preventive measures of the pandemic COVID-19. The odd of the likelihood of fail applying the basic preventive measures among farmers was 4 times higher as compared with the private business employees (AOR: 4.1; 95%CI: 1.25, 13.35). Farmers did not adopt

recommended measures to promote safe and consistent use of preventive measures, lock down to sustain their life and did not use face mask (which became uncomfortable during farming) [23].

Individuals currently not married were 2 times more likely not to apply the basic preventive measures as compared with currently married (AOR: 2.2; 95%CI: 1.24, 4.06). This might be due to the reason that, single individuals are less likely to have family support and who did not be able to stay at home to win their daily lives as compared with the married one.

The odd of the likelihood of the applicability of the basic preventive measures among respondents who have a family size 1-3 is 6 times higher as compared with those who have more than six families (AOR: 6.5; 95%CI: 3.21, 13.35) and residents who have a family size 4-6 were 2 times more; likely to fail to apply the basic preventive measures of the pandemic COVID-19 as compared with those who have more than six families (AOR: 2.4; 95%CI: 1.35, 4.25). This might be due to that having increased number of family members have better encouragement and awareness acquired through different sources. Information missed through one member can be acquired through the other member, which can be an input for the applicability of the preventive measures of the pandemic COVID-19.

Respondents who did not have any diagnosed medical health problems were 6 times more likely fail to apply the basic preventive measures of the pandemic COVID-19 (AOR: 6.4; 95%CI: 3.85, 10.83). Patients who have an experience of infection of chronic diseases have more compliance with preventive behaviors and are more careful in the prevention of diseases [20]. In addition, it might be due to that, individuals who have chronic diseases have information about its feature of being serious illness on those who have a chronic diseases and elder populations.

Knowledge became one of the associated factors for the applicability of the basic prevention measures of the pandemic COVID-19. Individuals who have poor knowledge were 3 times more likely to fail to apply the basic preventive measures of the pandemic COVID-19 (AOR: 3.5; 95%CI: 1.60, 7.40). This is due to knowledgeable individuals are more careful and responsible for the application of the preventive measures. In addition, knowledgeable individuals know the way of prevention, mode of transmission and the risk if it was not prevented appropriately.

Conclusion

Despite the application of preventive measures and vaccine delivery, the applicability of the pandemic COVID-19 preventive measures was too low, which indicate that the Zone is at risk for the infection. Rural residents, those who have lower educational level, farmers, non-marrieds, those who have lower family size, those who have diagnosed medical illnesses and those who have poor knowledge were prone to the infection with the pandemic COVID-19 due to the lower practice of applying the basic preventive measures. In addition, awareness creation should be in practice at all levels of the community especially lower educational classes and rural residents.

Recommendations

- Awareness creation should be in practice at for all the community especially lower educational classes.
- Individuals who have lower family size should be highly followed and a follow up link should be created between them and health extension practitioners and health facilities.
- Especial emphasis should be given for the rural residents to increase their practice level.
- Should supply the basic preventive measures such as mask and sanitizers for financially poor individuals
- Should strictly follow and encourage the rural residents.
- Must work more than the usual to increase the awareness of the residents.
- Residents must practice the information gained through different sources.
- Individuals who have no diagnosed medical illnesses should apply the basic preventive measures.

Acronyms and abbreviations

AOR: Adjusted odd ratio

CDC: Center for disease control

CI: Confidence interval

COR: Crude odd ratio

COVID-19: Corona Virus Disease 2019

FMOH: Federal Ministry of health

SARS-CoV-2: Severe acute respiratory syndrome coronavirus 2

SNNPR: South Nation Nationalities and Peoples Regional state

SPSS: Statistical package for social science

RNA: Ribo nucleic acid

WHO: World Health Organization

Declarations

Consent for publication

N/A

Availability of data and materials

All relevant data are within the manuscript and its Supporting Information files.

Competing interest

I declare that the authors have no any conflict of interest.

Funding

Not applicable

Author’s contribution

Samuel Dessu and Tadesse Tsehay were involved in the conceptualization, data curation, and formal analysis, funding acquisition, investigation, methodology. Tadele Girum, Abebe Timerga, Baye Tsegaye, Mamo Solomon and Abdurezak Kemal were involved in project administration, resource, software, validation. Mulugeta Geremew, Biru Migora, Yibeltal Mesfin, Fisha Alebel were involved in visualization and writing the original draft. Omega Tolosa, Shegaw Tesfa and Fedila Yasin were involved in the supervision, writing - review and editing. All authors reviewed the manuscript.

Author’s information

Samuel Dessu: Department of Public Health, College of Medicine and health Sciences, Wolkite University, Wolkite, Ethiopia**.** Tadesse Tsehay: Department of Nursing, College of Medicine and health Sciences, Wolkite University, Wolkite, Ethiopia**.** Tadele Girum: Department of Public Health, College of Medicine and health Sciences, Wolkite University, Wolkite, Ethiopia**.** Abebe Timerga: Department of Biomedical Sciences, College of Medicine and health Sciences, Wolkite University, Wolkite, Ethiopia**.** Mamo Solomon: Department of Nursing, College of Medicine and health Sciences, Wolkite University, Wolkite, Ethiopia**.** Baye Tsegaye: Department of Nursing, College of Medicine and health Sciences, Wolkite University, Wolkite, Ethiopia**.** Mulugeta Geremew: Department of Statistics, College of Natural and Computational Sciences, Wolkite University, Wolkite, Ethiopia**.** Biru Migora: Department of Statistics, College of Natural and Computational Sciences, Wolkite University, Wolkite, Ethiopia**.** Yibeltal Mesfin: Department of Midwifery, College of Medicine and health Sciences, Wolkite University, Wolkite, Ethiopia**.** Abdurezak Kemal: Department of Public Health, College of Medicine and health Sciences, Wolkite University, Wolkite, Ethiopia**.** Fisha Alebel: Department of Nursing, College of Medicine and health Sciences, Wolkite University, Wolkite, Ethiopia**.** Omega Tolosa: Department of Nursing, College of Medicine and health Sciences, Wolkite University, Wolkite, Ethiopia**.** Shegaw Tesfa: Department of Nursing, College of Medicine and health Sciences, Wolkite University, Wolkite, Ethiopia

Acknowledgements

We would like to thank the mothers who are directly involved in the study and administrator of each hospital for their effort and permission to conduct the study.

Corresponding Author

Correspondence to Samuel Dessu

References

1. Zhu, H., Wei, L. & Niu, P. The novel coronavirus outbreak in Wuhan, China. glob health res policy 5, 6 (2020).
2. World Health organization: Coronavirus disease 2019 (COVID-19) Situation Report – 94; 2020.
3. Ebrahim SH, Ahmed QA, Gozzer E, Schlagenhauf P, Memish ZA. Covid-19 and community mitigation strategies in a pandemic. BMJ. 2020;368.
4. UNICEF: for every child: Everything you need to know about washing your hands to protect against coronavirus (COVID-19); 2020.
5. Roba HS, Beyene AS, Mengesha MM, Ayele BH. Prevalence of Hypertension and Associated Factors in Dire Dawa City, Eastern Ethiopia: A Community-Based Cross-Sectional Study. International Journal of Hypertension. 2019;2019:9878437.

6. Wang C, Pan R, Wan X, Tan Y, Xu L, Ho CS. Immediate Psychological Responses and Associated Factors during the Initial Stage of the 2019 Coronavirus Disease (COVID-19) Epidemic among the General Population in China. Int J Environ Res Public Health. 2020;17(1729).
7. Huang C, Wang Y, Li X, Ren L, Zhao J, Hu Y, et al. Clinical features of patients infected with 2019 novel coronavirus in Wuhan, China. The Lancet. 2020;395:497-506.
8. Varshney M, Parel JT, Raizada N, Sarin SK. Initial psychological impact of COVID-19 and its correlates in Indian Community: An online (FEEL-COVID) survey. PLOS ONE. 2020;15(5):1-10.
9. M Z, Zhou M, Tang F, Wang Y, Nie H, Zhang L, et al. Knowledge, attitude, and practice regarding COVID-19 among healthcare workers in Henan, China. J Hosp Infect. 2020;105:183-7.
10. Gao H, Rujun H, Ling Y, Xiaoli Y, Hao T, Lan L, et al. Knowledge, attitudes and practices of the Chinese public with respect to coronavirus disease (COVID-19): an online crosssectional survey. BMC Public Health. 2020;20(1816).
11. World Health Organization: Ethiopia. 2.2 million COVID-19 vaccines allocated by the COVAX Facility arrive in Ethiopia, marking the start of the country’s COVID-19 vaccination campaign: 2021.
12. Kebede Y, Yitayih Y, Birhanu Z, Mekonen S, Ambelu A. Knowledge, perceptions and preventive practices towards COVID-19 early in the outbreak among Jimma university medical center visitors, Southwest Ethiopia. PLOS ONE. 2020;15(5).
13. Cirrincione L, Plescia F, Ledda C, Rapisarda V, Martorana D, Moldovan RE, et al. COVID-19 Pandemic: Prevention and Protection Measures to Be Adopted at the Workplace. Sustainability. 2020;12:3603.
14. Nira T, Punam R, Siddhartha D, Binod S, Bikash S, Prajjwal P, et al. COVID-19: a National Survey on perceived level of knowledge, attitude and practice among frontline healthcare Workers in Nepal. BMC Public Health. 2020;20(1905).
15. Akalu Y, Ayelign B, Molla MD. Knowledge, Attitude and Practice Towards COVID-19 Among Chronic Disease Patients at Addis Zemen Hospital, Northwest Ethiopia. Infection and Drug Resistance. 2020;13:1949—60.
16. Shibabaw T, Teferi B. Knowledge and Practice Toward Prevention of SARS-CoV-2 Among Healthcare Workers at Delghi Primary Hospital During a Massive Test Campaign in Northwest Gondar, Ethiopia: Institution-Based Descriptive Cross-Sectional Survey. Infection and Drug Resistance. 2021;14:381—90.
17. Mulu GB, Kebede WM, Worku SA, Mittiku YM, Ayelign B. Preparedness and Responses of Healthcare Providers to Combat the Spread of COVID-19 Among North Shewa Zone Hospitals, Amhara, Ethiopia, 2020. Infection and Drug Resistance. 2020;13:3171-8.
18. Olum R, Chekwech G, Wekha G, Nassozi DR, Bongomin F. Coronavirus Disease-2019: Knowledge, Attitude, and Practices of Health Care Workers at Makerere University Teaching Hospitals, Uganda. Front Public Health. 2020;8(181).
19. Ferdous MZ, Islam MS, Sikder MT, Mosaddek ASM, Zegarra-Valdivia JA, Gozal D. Knowledge, attitude, and practice regarding COVID-19 outbreak in Bangladesh: An onlinebased cross-sectional study. PLoS ONE. 2020;15(10):e0239254.

20. Zeinab AK, Wael AB, Shimaa KZ, Mohammed GH, Safa HA, Enas Z, et al. Knowledge, Attitude and Practice toward COVID-19 among Egyptians. Journal of Epidemiology and Global Health. 2020;10(4).
21. Feng J, Li TG, Qin PZ, Lu JY, Chen C, Chen ZQ, et al. Telephone survey and analysis of flu among citizens in Guangzhou. S China J Prev Med. 2013;39(4):35-7.
22. Xu YF, Li P, Zhou J, Guo LF. Investigation on cognitive behavior and psychological status of SARS in residents of Nanning city. Pract Prev Med. 2005;12(1):134-5.
23. Bonnie JM, Catherine BH. Health hazaeds and preventive measures of farm women. AAOHN Journal. 2002;50(7).

Table legends

Table 1: The Socio-demographic characteristics of residents in Guraghe Zone, 2020 (n=634)

Table 2: Knowledge status of the respondents on COVID-19 among residents in Guraghe Zone, 2020 (n=634)

Table 3 : The level of attitude towards the prevention of COVID-19 among residents in Guraghe Zone, 2020 (n=634)

Table 4: The applicability of the basic preventive measures of the pandemic COVID-19 among residents in Guraghe Zone, 2020 (n=634)

Table 5: Bivariable analysis of factors associated with the applicability of the basic preventive measures of the pandemic COVID-19 among residents in Guraghe Zone, 2020 (n=634)

Table 6: Factors associated with the applicability of the basic preventive measures of the pandemic COVID-19 among residents in Guraghe Zone, 2020 (n=634)

Table 1:

Variables	Category	Frequency	Percent
Age (in years)	18-35	205	32.3
	36-64	313	49.4
	>64	116	18.3
Residence	Urban	142	22.4
	Rural	492	77.6
Educational status	Unable to read and write	51	8.0
	Only read and write	59	9.3
	Grade 1-8	121	19.1
	Grade 9-12	131	20.7
	College and above	272	42.9
Occupational status	Farmer	83	13.1
	Student	160	25.2
	Currently not employed	37	5.8
	Government employed	248	39.1
	Private business employed	106	16.7
Religious status	Orthodox	378	59.6
	Muslim	162	25.6
	Protestant	60	9.5
	Catholic	26	4.1
	Others	8	1.3
Family size	1-3	205	32.3
	4-6	313	49.4
	>6	116	18.3

Table 2

Knowledge assessment questions	Knowledge Category		
	Yes	No	I don't know
**Knowledge of cause and symptoms**			
The main clinical symptoms of COVID-19 are fever, fatigue, dry cough, and myalgia	606(95.6%)	14(2.2%)	14(2.2%)
Unlike the common cold, stuffy nose, runny nose, and sneezing are less common in persons infected with the COVID-19 virus	261(41.2%)	332(52.4%)	41(6.5%)
**Knowledge of high risk and prognosis**			
Not all persons with COVID-19 will develop severe cases. Only those who are elderly, have chronic illnesses & are obese are more likely to be severe cases	246(38.8%)	350(55.2%)	38(6.0%)
There currently is no effective cure for COVID-19, but early symptomatic and supportive treatment can help most patients recover from the infection	188(29.7%)	437(68.9%)	9(1.4%)
**Knowledge about mode of transmission and infectiousness**			
The COVID-19 virus spreads via respiratory droplets of infected individuals	541(85.3%)	82(12.9%)	11(1.7%)
Eating or contacting wild animals would result in the infection by the COVID-19 virus	416(65.6%)	154(24.3%)	64(10.1%)
Persons with COVID-19 cannot infect the virus to others when a fever is not present	112(17.7%)	489(77.1%)	33(5.2%)
**Knowledge about ways of prevention**			
Proper washing hand with soap and water is one method of preventing COVID-19	594(93.7%)	40(6.3%)	0(0%)
One way of prevention of COVID-19 is not touching the eye, nose by unwashed hands	601(94.8%)	33(5.2%)	0(0%)
To prevent the infection by COVID-19, individuals should	542(85.5%)	89(14.0%)	3(0.5%)

avoid going to crowded places such as train stations, market and avoid taking public transportations			
Ordinary residents can wear general medical masks to prevent the infection by the COVID- 19 virus	593(93.5%)	35(5.5%)	6(0.9%)
People who have contact with someone infected with the COVID-19 virus should be immediately isolated in a proper place	563(88.8%)	69(10.9%)	2(0.3%)
Isolation and treatment of people who are infected with the COVID-19 virus are effective ways to reduce the spread of the virus	596(94.0%)	34(5.4%)	5.4%)
Children and young adults don’t need to take measures to prevent the infection by the COVID-19 virus	106(16.7%)	501(79.0%)	27(4.3%)

Table 3

Attitude domain	Level of attitude				
	Strongly disagree	Disagree	Neutral	Agree	Strongly agree
Do you agree that the Ethiopian government was handling the COVID-19 health crisis well?	52(8.2%)	126(19.9%)	37(5.8%)	364(57.4%)	55(8.7%)
Do you agree that COVID-19 will be successfully controlled?	37(5.8%)	87(13.7%)	65(10.3%)	391(61.7%)	54(8.5%)
Do you agree that Ethiopian would be able to win the battle against COVID-19?	24(3.8%)	38(6.0%)	31(4.9%)	425(67.0%)	116(18.3 %)
Do you agree that Self-protection is necessary for the protection of others?	8(1.3%)	18(2.8%)	12(1.9%)	402(63.4%)	194(30.6 %)
Do you agree that Lockdown is an effective measure to control the transmission?	27(4.3%)	59(9.3%)	20(3.2%)	334(52.7%)	194(30.6 %)
Do you agree that wearing face mask protects against COVID-19?	3(0.5%)	31(4.9%)	5(0.8%)	421(66.4%)	174(27.4 %)
Do you agree that appropriate hand washing practice is important in the prevention of COVID-19?	5(0.8%)	17(2.7%)	6(0.9%)	409(64.5%)	197(31.1 %)
Do you agree that the transmission of COVID-19 can be minimized in keeping physical distance?	17(2.7%)	37(5.8%)	14(2.2%)	386(60.9%)	180(28.4 %)
Did you agree that COVID-19 leads to stigma?	45(7.1%)	75(11.8%)	21(3.3%)	369(58.2%)	124(19.6 %)
Do you agree that if getting COVID-19, you will accept isolation in	30(4.7%)	67(10.6%)	14(2.2%)	344(54.3%)	179(28.2

health facilities?					%)

Table 4

Variables	Applicability of preventive measures	
	**Yes**	**No**
Lockdown	91(14.4%)	543(85.6%)
Appropriate frequent hand washing	253(39.9%)	381(60.1%)
Stop hand shaking	152(24.0%)	482(76.0%)
Avoid touching eye, nose, and mouth	110(17.4%)	524(82.6%)
Using face mask as a preventive measure	88(13.9%)	546(86.1%)
Apply two meters physical distance	63(9.9%)	571(90.1%)
Avoided going to the crowed place	79(12.5%)	555(87.5%)
Using elbow to cover during coughing/sneezing	454(71.6%)	180(28.4%)

Table 5

Variables	Category	Status		COR (95%CI)
		Not yet applied basic preventive measures	Applied basic preventive measures	
Age (in years)	18-35	349	68	2.2(0.55, 8.72)
	36-64	166	41	1.7(0.43, 7.00)
	>64	7	3	1
Sex	Male	267	53	1
	Female	255	59	0.85(0.57, 1.29)
Residence	Urban	124	18	1
	Rural	398	94	0.62(0.36, 1.06)*
Educational level	Unable to read and write	47	4	2.08(0.71, 6.10)*
	Only read and write	52	7	1.32(0.56, 3.10)*
	Grade 1-8	90	31	0.51(0.30, 0.87)*
	Grade 9-12	102	29	0.62(0.37, 1.06)*
	College and above	231	41	1
Occupational status	Farmer	78	5	3.62(1.29, 10.13)*
	Student	125	35	0.83(0.45, 1.54)
	Currently not employed	28	9	0.72(0.29, 1.77)
	Government employed	205	43	1.2(0.62, 1.99)
	Private business employed	86	20	1
Marital status	Currently married	284	78	1
	Currently not married	238	34	1.9(1.24, 2.98)*
Family size	1-3	188	17	6.0(3.23, 11.30)*
	4-6	259	54	2.62(1.62, 4..24)*
	>6	75	41	1
Mass media availability	Yes	445	81	1
	No	77	31	0.45(0.28, 0.73)*
Diagnosed medical health problem	Yes	82	56	1
	No	440	56	5.36(3.46, 8.32)*

Knowledge status	Good	127	12	1
	Poor	395	100	0.37(1.19, 0.70)*
Attitude	Favorable	33	4	1
	Unfavorable	489	108	0.55(0.19, 1.58)
*Key note: *indicates variables which have p-value <0.25 in bivariable analysis*				

Table 6

Variables	Category	COR (95%CI)	AOR(95%CI)	P-value
Residence	Urban	1	1	0.0047
	Rural	0.62(0.36, 1.06)*	4.78(2.5, 8.9)**	
Educational level	Unable to read and write	2.08(0.71, 6.10)*	1.03(0.28, 3.88)	0.011
	Only read and write	1.32(0.56, 3.10)*	0.42(0.13, 1.37)	
	Grade 1-8	0.51(0.30, 0.87)*	3.7(1.70, 7.90)**	
	Grade 9-12	0.62(0.37, 1.06)*	0.55(0.27, 1.14)	
	College and above	1	1	
Occupational status	Farmer	3.62(1.29, 10.13)*	4.1(1.25, 13.35)**	0.027
	Student	0.83(0.45, 1.54)	0.45(0.21, 1.01)	
	Currently not employed	0.72(0.29, 1.77)	0.57(0.0.19, 1.72)	
	Government employed	1.2(0.62, 1.99)	0.47(0.19, 1.01)	
	Private business employed	1	1	
Marital status	Currently married	1	1	0.003
	Currently not married	1.9(1.24, 2.98)*	2.2(1.24, 4.06)**	
Family size	1-3	6.0(3.23, 11.30)*	6.5(3.21, 13.35)**	0.0001
	4-6	2.62(1.62, 4..24)*	2.4(1.35, 4.25)**	
	>6	1	1	
Diagnosed medical health problem	Yes	1	1	0.0001
	No	5.36(3.46, 8.32)*	6.4(3.85, 10.83)**	
Knowledge status	Good	1	1	0.002
	Poor	0.37(1.19, 0.70)*	3.5(1.60, 7.40)	
*Key note: *indicates variables which have p-value <0.25, **indicates variables which have p-value <0.05*				

Response to reviewers

Reviewer 1:

Line space in abstract part is not consistent.so; make it consistent the line space for the conclusion part is not consistent with the other part.

- **Response**: it as revised and all the part have similar spacing.

Reviewer 2:

The author addresses all the comments, however, still they did not understood one of my comment regarding redundancy of the sentence the conclusion in both the abstract and the main body. Therefore; please rephrase and rewrite the conclusion to minimize repetition.

- **Response**: It was revised and corrected as: - "Despite the application of preventive measures and vaccine delivery, the applicability of the pandemic COVID-19 preventive measures was too low, which indicate that the Zone is at risk for the infection. Rural residents, those who have lower educational level, farmers, non-marrieds, those who have lower family size, those who have diagnosed medical illnesses and those who have poor knowledge were prone to the infection with the pandemic COVID-19 due to the lower practice of applying the basic preventive measures. In addition, awareness creation should be in practice at all levels of the community especially lower educational classes and rural residents.
