# Supplementary material for: The applicability of basic preventive measures of the pandemic COVID-19 and associated factors among residents in Guraghe Zone
Source: PLoS One. 2021 Aug 25;16(8):e0256598. doi: 10.1371/journal.pone.0256598 (PMC8386865; doi:10.1371/journal.pone.0256598)
Supplement: Attachment — Submitted filename: Response to reviewers.docx [file pone.0256598.s003.docx]

**Response to the Reviewers**

1. Is the manuscript technically sound, and do the data support the conclusions?

The manuscript must describe a technically sound piece of scientific research with data that supports the conclusions. Experiments must have been conducted rigorously, with appropriate controls, replication, and sample sizes. The conclusions must be drawn appropriately based on the data presented.

Reviewer #1: Yes

Reviewer #2: Yes

Reviewer #3: No

Response; Thank you and certain revision were made.

2. Has the statistical analysis been performed appropriately and rigorously?

Reviewer #1: No

Reviewer #2: Yes

Reviewer #3: No

Response: Thank you and it was revised in detail.

3. Have the authors made all data underlying the findings in their manuscript fully available?

The [PLOS Data policy](http://www.plosone.org/static/policies.action#sharing) requires authors to make all data underlying the findings described in their manuscript fully available without restriction, with rare exception (please refer to the Data Availability Statement in the manuscript PDF file). The data should be provided as part of the manuscript or its supporting information, or deposited to a public repository. For example, in addition to summary statistics, the data points behind means, medians and variance measures should be available. If there are restrictions on publicly sharing data—e.g. participant privacy or use of data from a third party—those must be specified.

Reviewer #1: Yes

Reviewer #2: Yes

Reviewer #3: Yes

Response: Thank you

4. Is the manuscript presented in an intelligible fashion and written in standard English?

PLOS ONE does not copyedit accepted manuscripts, so the language in submitted articles must be clear, correct, and unambiguous. Any typographical or grammatical errors should be corrected at revision, so please note any specific errors here.

Reviewer #1: No

Reviewer #2: Yes

Reviewer #3: Yes

Response: Thank you and it was revised by a language expert.

5. Review Comments to the Author

Please use the space provided to explain your answers to the questions above. You may also include additional comments for the author, including concerns about dual publication, research ethics, or publication ethics. (Please upload your review as an attachment if it exceeds 20,000 characters)

**Response**: It was revised and we have responded point by point for each raised concerns and corrected as highlighted in the revised version.

Reviewer #1:

1. The manuscript has a lot of typo errors grammatical, spelling, punctuation, and consistency in word usage like; COVID-19, COVID-2019 and COVID 19, Guraghe zone and Gurage zone, dyed instead of died.

- **Response**: Thank you. All the inconsistencies were resolved and the whole manuscript was revised by language expert.

2. High knowledge: If the respondent answers 11 of the 14 knowledge assessment questions correctly (10). What if the respondent answer 12, 13, and 14 of 14 knowledge assessment questions correctly????

**Response**: Thank you and it was revised and it was to mean at least 11 of the 14 assessment questions.

3. Moderate knowledge: If the respondent answers nine of the 14 knowledge assessment questions correctly (10). What if the respondent answers 10 of 14 knowledge assessment questions correctly????

- **Response**: It was revised and which was to mean at least nine of the knowledge assessment questions.

4. Poor knowledge: If the respondent responds below nine knowledge assessment questions correctly (10).

- **Response**: It was revised as:- “Poor knowledge: If the respondent responds ≤ 8 knowledge assessment questions correctly”.

5. Good knowledge: If the respondent responds above nine knowledge assessment questions correctly

- **Response**: It was revised as: - “Good knowledge: If the respondent responds ≥ 9 knowledge assessment questions correctly”.

6. How did you measure attitude is not operationalized

- **Response**: It was mentioned at the end of data collection tool and procedure but no we have stated at the subtitle, operational definition.

7. The maximum total score ranged from 0–13, with a higher score indicating better knowledge about COVID-19. How can it be the maximum score of 13 if one individual answers all 14 questions correctly the maximum score will be 14? So, how do you justify it?

- **Response**: Thank you. It was a typing error, which was to mean 14. If a respondent answers all the knowledge assessment questions correctly, stated as scored 14 of the 14 questions.

8. Data were cleaned, edited, coded, and entered into Epi-data version 3.1 and exported to SPSS version 25 for Windows. How can you cleaned, edited, and coded before data entry?

**Response**: The statement was rephrased and stated as:- “Data were entered into Epi-data version 3.1 and exported to SPSS version 25 for Windows, then cleaned, edited, coded and exploratory data analysis was carried out to check the levels of missing values, presence of influential outliers, multi-co linearity”.

9. Television is not social media?

**Response**: It was revised and which was to mean mass media.

10. How do you manage the multicollinearity between being a rural residence and a lower educational level?

**Response**: All the variables were checked for multicholinearity but multicholinearity was not existed. Therefore; no any management is required unless multicholinearity was observed.

11. Should supply the basic preventive measures such as mask and sanitizers for financially poor individuals: You didn’t assess the availability of these preventive accessories so how can you recommend.

**Response**: It was missed during manuscript preparation and incorporated now. This study was conducted as a baseline for further studies and for conducting community service across the study area. This study was presented within the university and community service was delivered through delivering the sanitizers, mask and health information dissemination.

12. Those independent variables that had p-value less than 0.25 in bivariate analysis were entered in to the multivariable logistic regressions model. What is your justification to use 0.25 as cut off points?

**Response**: In this study, we had used 12 independent variables. If we had sufficient variables, we can minimize the cutoff point but if the number of variables were not much we can increase the cutoff point. In addition; increasing the cutoff point will keep the marginally significant variables. Therefore; we have used the cutoff point 0.25 to select the candidate variable for multivariable analysis.

Reviewer #2:

In this manuscript, the authors conducted a community based cross-sectional study to assess The applicability of basic preventive measures of the pandemic COVID-19 and associated factors among residents in Guraghe Zone. The study is well written, is easy to follow and covers a hot topic, but some issues should be improved before publication.

1) The study is well thought off. I believed that the topic and the content of the manuscript was different. So, it will be advisable to modify the title like KAP

**Response**: It was not aimed to investigate the knowledge and attitude but both of them were independent variables. They were described and as independent factors associated with the applicability of the basic preventive measures of COVID-19. As you have seen in the introduction part, it was focused to show the gap in the applicability of the basic preventive measures. In addition, as you have seen the outcome variable is the applicability of the basic preventive measures of the pandemic COVID-19, not KAP.

2) In the method section, replace Method by Method and material

**Response**: It was replaced accordingly.

3) There are many language mistakes, please revisit the manuscript for correction.

**Response**: The whole manuscript was revised by a language expert.

4) Reference for your operational definition?

**Response**: Thank you all the operational definitions were cited.

5) Please give some explanations about the current availability of the vaccine

**Response**; It was incorporated at the introduction section.

6) Please complete all necessary information on the title of each Table

**Response**: Thank you. It was revised and all the necessary information was incorporated.

7) Discussion section: Will be useful to the reader to add some interesting recent literature about the updates against COVID-19 outbreak and related tools to counteract the same

**Response**: Certain updated recent articles were cited.

8) Used very few reference, which results poor interpretation of your result, please use the following reference (Akalu Y, Ayelign B, Molla MD. Knowledge, attitude and practice towards COVID-19 among chronic disease patients at Addis Zemen Hospital, Northwest Ethiopia. Infection and drug resistance. 2020;13:1949., Mulu GB, Mittiku YM, Jemere BA. Preparedness and Approaches of Healthcare Providers to Tackle the Transmission of Covid-19 among North Shewa Zone Hospitals, Amhara, Ethiopia, 2020.,Shibabaw T, Teferi B. Knowledge and Practice Toward Prevention of SARS-CoV-2 Among Healthcare Workers at Delghi Primary Hospital During a Massive Test Campaign in Northwest Gondar, Ethiopia: Institution-Based Descriptive Cross-Sectional Survey. Infection and Drug Resistance. 2021;14:381. )

**Response**: Thank you. All of them were cited.

9) Conclusion Section: The paragraph requires a general revision to eliminate redundant sentences and please refine and don’t repeat it in the abstract part.

**Response**: The conclusion was revised and certain amendments were made.

Response to Reviewer #3

Why use design effect 1.5? , is there scientifically recommended to use design effect 1.5

- **Response**: Design effect is determined by the researcher in considering the heterogeneity of the population. Most researchers use design effect 2 but it is possible to use also 1.5. We have decided to use it 1.5 in considering, the heterogeneity of the populations (which is adequate for them, there is no extreme heterogeneity between them) and the cost that we have afford.

Minor

1. There is sentence and paragraph without reference on introduction part

**Response**: Thank you. All the statements were cited.

2. On table 5 there is missing data , please incorporate this data

**Response**: It was not a missing data, which was for the variables which were not statistically significant in multivariable analysis but to resolve this, we have the table in to two (table 5 & 6).

3. It is better to exclude those data are not significant on multi-variate regression

**Response**: It was revised and removed.

4. Please include the p-value for those factors that are significant at multi-variant

**Response**: Thank you, it was included.
