# Supplementary material for: The applicability of basic preventive measures of the pandemic COVID-19 and associated factors among residents in Guraghe Zone
Source: PLoS One. 2021 Aug 25;16(8):e0256598. doi: 10.1371/journal.pone.0256598 (PMC8386865; doi:10.1371/journal.pone.0256598)
Supplement: Attachment — Submitted filename: Response to reviewers.docx [file pone.0256598.s004.docx]

**Response to reviewers**

Reviewer 1:

Line space in abstract part is not consistent.so; make it consistent the line space for the conclusion part is not consistent with the other	part.

- **Response**: it as revised and all the part have similar spacing.

Reviewer 2:

The author addresses all the comments, however, still they did not understood one of my comment regarding redundancy of the sentence the conclusion in both the abstract and the main body. Therefore; please rephrase and rewrite the conclusion to minimize repetition.

- **Response**: It was revised and corrected as: - “Despite the application of preventive measures and vaccine delivery, the applicability of the pandemic COVID-19 preventive measures was too low, which indicate that the Zone is at risk for the infection. Rural residents, those who have lower educational level, farmers, non-marrieds, those who have lower family size, those who have diagnosed medical illnesses and those who have poor knowledge were prone to the infection with the pandemic COVID-19 due to the lower practice of applying the basic preventive measures. In addition, awareness creation should be in practice at all levels of the community especially lower educational classes and rural residents.
