# Supplementary material for: The applicability of basic preventive measures of the pandemic COVID-19 and associated factors among residents in Guraghe Zone
Source: PLoS One. 2021 Aug 25;16(8):e0256598. doi: 10.1371/journal.pone.0256598 (PMC8386865; doi:10.1371/journal.pone.0256598)
Supplement: Attachment — Submitted filename: Comments to Autor.docx [file pone.0256598.s006.docx]

**Comments**

The authors have carefully addressed almost all the issues raised by reviewers in the first review process. However, the additional comments below should be addressed to enhance the quality of the paper.

**On Abstract, data processing and analysis, and result session better to address these comments which are highlighted in the main manuscript:**

- P-value ≤0.05 better to change <0.05
- All prevalence, proportion, and magnitude are better to be in one decimal point including its confidence intervals. For instance, 21.9% (95%CI: 18.9, 25.1) of the respondents have good knowledge, 94.2% (95%CI: 92.3, 95.9) had a favorable attitude, and 17.7% (95% CI: 14.7, 20.5) apply basic preventive measures …………
- The odds ratio and its respective confidence interval better to be in two decimal points. For example, (AOR: 4.78; 95%CI: 2.50, 8.90)
- As a principle; the prevalence, proportion, magnitude, odds, and its respective confidence interval should have a similar decimal points which means one decimal point for prevalence, proportion, and magnitude and two decimal points for odds.
- All the recommendations have no owner it doesn’t tell anything about for whom you are going to recommend. So, it is better to indicate the specific stakeholders for each of your recommendations.
