# Supplementary material for: The applicability of basic preventive measures of the pandemic COVID-19 and associated factors among residents in Guraghe Zone
Source: PLoS One. 2021 Aug 25;16(8):e0256598. doi: 10.1371/journal.pone.0256598 (PMC8386865; doi:10.1371/journal.pone.0256598)
Supplement: Attachment — Submitted filename: Response to reviewers.docx [file pone.0256598.s007.docx]

**Response to reviewers**

- P-value ≤0.05 better to change <0.05

Response: It was revised and corrected	as	“P-value <0.05 was used as a cutoff point to determine statistical significance in multiple logistic regressions for the final model”.

- All prevalence, proportion, and magnitude are better to be in one decimal point including its confidence intervals. For instance, 21.9% (95%CI: 18.9, 25.1) of the respondents have good knowledge, 94.2% (95%CI: 92.3, 95.9) had a favorable attitude, and 17.7% (95% CI: 14.7, 20.5) apply basic preventive measures …………

Response: It was revised and corrected all over the manuscript as:- “In this study, 17.7% (95% CI: 14.7, 20.5) of the respondents apply the basic preventive measures towards the prevention of the pandemic COVID-19”.

- The odds ratio and its respective confidence interval better to be in two decimal points. For example, (AOR: 4.78; 95%CI: 2.50, 8.90)
  - Response: It was revised and corrected as:- “In addition, being rural resident (AOR: 4.78,; 95%CI: 2.50, 8.90), being studied grade 1-8 (AOR: 3.70; 95%CI: 1.70, 7.90), being a farmer (AOR: 4.10; 95%CI: 1.25, 13.35), currently not married (AOR: 2.20, 95%CI: 1.24, 4.06), having family size 1-3(AOR: 6.50; 95%CI: 3.21, 3.35), have no diagnosed medical illness (AOR: 6.40; 95%CI: 3.85, 10.83) and having poor knowledge (AOR: 3.50; 95%CI: 1.60, 7.40) were factors which are statistically significant in multivariable logistic regression model”.
- As a principle; the prevalence, proportion, magnitude, odds, and its respective confidence interval should have a similar decimal points which means one decimal point for prevalence, proportion, and magnitude and two decimal points for odds.
  - Response: It was revised and corrected as per your recommendations.
- All the recommendations have no owner it doesn’t tell anything about for whom you are going to recommend. So, it is better to indicate the specific stakeholders for each of your recommendations.
  - Response: It was revised and the recommendations were given for the specific bodies.
